# Synthesis of 1-Substituted Carbazolyl-1,2,3,4-tetrahydro- and Carbazolyl-3,4-dihydro-β-carboline Analogs as Potential Antitumor Agents

**DOI:** 10.3390/md9020256

**Published:** 2011-02-10

**Authors:** Ya-Ching Shen, Yao-To Chang, Chun-Ling Lin, Chia-Ching Liaw, Yao Haur Kuo, Lan-Chun Tu, Sheau Farn Yeh, Ji-Wang Chern

**Affiliations:** 1 School of Pharmacy, National Taiwan University, Taipei 100, Taiwan; Email: ato@kiss99.com (Y.-T.C.); 6850101@gmail.com (C.-L.L.); biogodas@hotmail.com (C.-C.L.); jwchern@ntu.edu.tw (J.-W.C.); 2 National Research Institute of Chinese Medicine, Taipei 112, Taiwan; Email: kuoyh@nricm.edu.tw; 3 Institute of Biochemistry, National Yang-Ming University, Taipei 112, Taiwan; Email: fyeh@ym.edu.tw (S.F.Y.)

**Keywords:** carbazolyl-1,2,3,4-tetrahydro-β-carbolines, carbazolyl-3,4-dihydro-β-carbolines, antitumor agents, structure activity relationship

## Abstract

A series of 1-substituted carbazolyl-1,2,3,4-tetrahydro- and carbazolyl-3,4-dihydro-β-carboline analogs have been synthesized and evaluated for antitumor activity against human tumor cells including KB, DLD, NCI-H661, Hepa, and HepG2/A2 cell lines. Among these, compounds **2**, **6**, **7**, and **9** exhibited the most potent and selective activity against the tested tumor cells. As for inhibition of topoisomerase II, compounds **1**–**14** and **18** showed better activity than etoposide. Among them, compounds **3**, **4**, **7**, **9**, and **10** exhibited potent activity. The structure and activity relationship (SAR) study revealed correlation between carbon numbers of the side chain and biological activities. The molecular complex with DNA for compound **2** was proposed.

## 1. Introduction

Marine invertebrates are rich in β-carboline alkaloids [1,2,3]. These natural β-carboline metabolites have been found to possess interesting antitumor and antiviral activities [4,5,6]. Eudistomins [7,8] and manzamines [9,10], which were isolated from tunicates and sponges, respectively, are of particular interest. The antiviral eudistomines C and E were active against HSV-2, Vaccinia virus and RNA viruses [11]. In addition, the novel manzamines exhibited potent antitumor, antibacterial, antifungal and anti-HIV activities [12,13,14]. The major compound, manzamine A, showed most potent activity against murine P-388 cells [15]. Our previous paper reported that manzamines A-D and H showed significant cytotoxicities against human KB-16, A-549 and HT-29 tumor cells [16]. The β-carboline and 3,4-dihydro-β-carboline moieties in manzamines appear to be essential for the biological activity. In order to investigate the structure and activity relationship (SAR) and bioactive center of manzamine A, the synthesis of 1-substituted carbazolyl-1,2,3,4-tetrahydro- and carbazolyl-3,4-dihydro-β-carboline derivatives were initiated [17]. The limited source of manzamine A make this current investigation more urgent and important. The purpose of this study does not include all SAR studies of manzamines and β-carboline analogs. However, a combination of β-carboline and various carbazole analogs is addressed. An attempt to realize the possible active center of manzamine A and the importance of the alkyl substituents on the nitrogen atom of carbazole ring was conducted. Thus, a facile synthetic method by the application of *N*-alkylation, Duff reaction [18], Pictet-Spengler reaction [19,20] and DDQ oxidation [21] succeeded in the production of compounds **1**-**18**. In this communication, we wish to report the preparation, structural elucidation and bioactivities of 1-substituted carbazolyl-1,2,3,4-tetrahydro- and carbazolyl-3,4-dihydro-β-carboline analogs.

## 2. Results and Discussion

### 2.1. Analog Design and Chemistry

**Figure 1:**
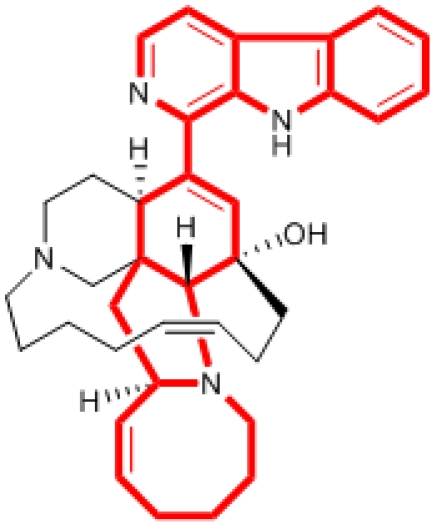
The main part of manzamine A.

**Scheme 1:**
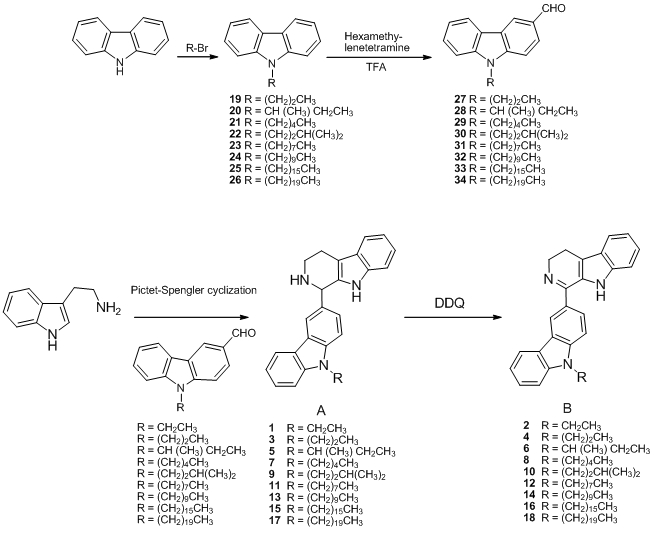
Preparation of compounds **1**-**36**.

The basic strategy for the synthesis of the target substances involved molecular modeling and SAR studies of manzamine analogs. To improve our knowledge of the main structural requirements needed for high antitumor activity, we synthesized two new series of 1-substituted carbazolyl-1,2,3,4-tetrahydro-β-carboline and carbazolyl-3,4-dihydro-β-carboline derivatives based on the analog design of manzamine A. These new compounds bear an *N*-alkyl carbazole conjugated with a β-carboline-like nucleus. The main part of manzamine A illustrated in Figure 1 is similar in both shape and size to our target compounds. The distances between *N* atom on carbazole and *N* atoms on carboline-nucleus are almost the same as those in the main part. With the aim of studying the SAR, we focused on the lengh of the *N*-alkyl side chain on the carbazole ring. Compounds **19**-**26** were prepared by *N*-alkylation of carbazole with the appropriate alkyl bromide as depicted in Scheme 1. Subsequent synthesis of compounds **27**-**34** was achieved by Duff reaction, which required hexamethylenetetramine/trifloroacetic acid and the appropriate *N*-alkyl carbazole. Compounds **1**, **3**, **5**, **7**, **9**, **11**, **13**, **15**, and **17** (A series) were furnished from tryptamine and series of *N*-substituted 3-carbazolyl carboxyaldehydes (**27**-**34**, and *N*-ethyl-3-carboxyaldehyde, which was purchased from Sigma-Aldrich Co.) byapplication of Pictet-Spengler cyclization. Subsequent oxidation of 1,2,3,4-tetrahydro-β-carbolines (A) by DDQ yielded 3,4-dihydro-β-carboline derivatives (B series: **2**, **4**, **6**, **8**, **10**, **12**, **14**, **16**, **18**). The preparation and spectral data for **1**-**34** are described in the Experimental Section.

### 2.2. Biological Activity

Cytotoxicity of new products **1**-**18** was tested against KB (human mouth epidermoid carcinoma); DLD (human colon adenocarcinoma), NCI-H661 (human lung large cell carcinoma) and Hepa (human hepatoma), HepG2/A2 (human hepatoma) tumor cells *in vitro*. The IC_50_ values of these compounds are summarized in Table 1. Table 1 shows that compounds **1**-**1** **2** exhibited significant and/or selective cytotoxic activities. Among them, compounds **2** and **6** are most potent against KB tumor cells selectively. Compound **7** is more potent than **2** against NCI-H661 tumor cells although compound **2** shows most promising activity against all tumor cells. On the other hand, compounds **14**-**18** are inactive toward four tumor cells while compounds **11**-**13** exhibit weak, marginal or no activity. Table 1 also shows that these compounds have selective cytotoxicity against HepG2/A2 tumor cells. Compounds **2**-**4** and **8** are active while others are inactive. In this assay, compound **2** exhibits most potent activity against the HepG2/A2 system.

**Table 1:** Cytotoxicity of Compounds **1**-**18** against Human Cancer Cells (IC_50_, μg/mL) ^a^.

Compound/Cell line	KB	DLD	NCI-H661	Hepa	HepG2/A2
1	3.47	1.69	1.82	NT ^b^	NT
2	0.71	1.09	0.84	NT	0.60
3	1.84	1.58	2.14	NT	6.10
4	2.72	2.16	5.02	NT	5.10
5	3.00	NT	NT	2.25	NT
6	0.48	NT	NT	2.67	NT
7	3.45	3.75	0.22	NT	20.50
8	>20	1.29	3.39	NT	5.40
9	2.96	NT	NT	1.12	NT
10	3.13	NT	NT	4.40	NT
11	>20	1.57	>20	NT	20.70
12	9.13	2.55	5.28	NT	30.10
13	6.36	10.40	13.90	NT	>60
14	>20	>20	>20	>20	>60
15	>20	>20	>20	>20	>60
16	>20	>20	>20	>20	>60
17	>20	>20	>20	>20	>60
18	>20	>20	>20	>20	>60

^a^ The concentration of compound which inhibited 50% (IC_50_) of the growth of tumor cell lines (KB, human mouth epidermoid carcinoma; DLD, human colon adenocarcinoma; NCI-H661, human lung large cell carcinoma; Hepa and HepG2/A2, human hepatoma); All data estimated by interpolation method; Doxorubicin was used as a positive control (IC_50_, 0.1 μg/mL); ^b^ Not tested.

The SAR study revealed that there was not a linear relationship between the carbon number of the side chain at *N* atom in carbazole and cytotoxicity. Nevertheless, we observed that, in general, elongation of the alkyl chain resulted in a decrease in activity. In fact, compounds **11**-**18** showed very weak or no activity toward HepG2/A2, KB and NCI-H661 cells even though compound **11** was active in DLD assay. 

Inhibition activity of compounds **1**-**18** was evaluated against human DNA topoisomerases I and II. Table 2 indicates that compounds **1**-**14** and **18** are more potent inhibitors of human DNA topoisomerase II than etoposide. Among the tested compounds, **3**, **4**, **7**, **9** and **10** were most potent. On the other hand, all compounds were inactive or showed mild activity against DNA topoisomerase I. Compounds **3**, **4**, **9** and **10** were 10- to 15-fold more potent against topoisomerase II (compared to etoposide) and superior to compounds **1** and **2**. On the other hand, compound **18** showed great activity while compounds **15**-**17** did not. 

Compound **7** showed much better activity than that of **2** suggesting that the different degrees of activity might be explained by the differences in binding affinity or bioavailability such as drug uptake and fate of metabolism.

**Table 2:** Inhibition of DNA topoisomerases I and II (IC_50_, μg/mL) ^a^ by compounds **1**-**18**.

Compound	DNA topoisomerase I	DNA topoisomerase II
1	>100	9.5
2	>100	8.5
3	47.0	1.6
4	>100	2.1
5	>100	10.0
6	>100	26.0
7	89.0	2.7
8	58.0	22.0
9	96.0	2.8
10	67.0	2.7
11	>100	30.0
12	>100	29.0
13	>100	28.0
14	>100	19.0
15	>100	>100
16	>100	>100
17	>100	>100
18	>100	5.6
Camptothecin	17.0	NT
Etoposide	NT	31.0

^a^ Each compound was examined with five concentrations at 5, 10, 25, 50 and 100 μg/mL; The IC_50_ value was measured based on the degree of inhibition at these five concentrations; NT: Not tested.

### 2.3. Molecular Modeling

**Figure 2:**
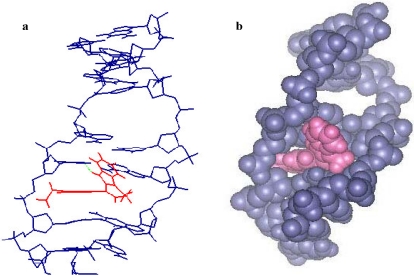
Plot of the d(CGCATGGG)_2_-compound **2** complex. (**a**) Line plot: Three-dimensional structural model of **2** with DNA receptor binding site; (**b**) Space-filling plot: Compound **2**, red; DNA duplex, blue.

**Figure 3:**
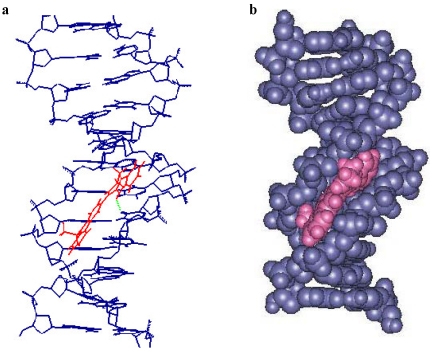
Plot of the d(CGCGAATTGCG)_2_-compound **2** complex. (**a**) Line plot: Three-dimensional structural model of **2** with DNA receptor binding site; (**b**) Space-filling plot: Compound **2**, red; DNA duplex, blue.

To rationalize the biological activity results obtained for this novel series of carbazolyl-3,4-dihydro-β-carbolines, we carried out a molecular modeling study of compound **2**. The molecular modeling was performed by making use of two double strand DNA fragments (CGCTAGGG)_2_ and (CGCGAATTCGCGG)_2_.  Figure 2 shows the result of d(CGCATGGG)_2_-compound **2** complex. It was found that the binding conformation of this complex was formed by intercalation. Unlike the usual planar intercalators, compound **2** inserts into base pairs of DNA in a scissor-like conformation. We observed that the carbazole moiety of compound **2** was parallel with the thymidine base. However, the conformation of d(CGCGAATTGCG)_2_-compound **2** complex, as illustrated in Figure 3, was formed by minor groove binding. In this case, compound **2** interacts with DNA by putting the carbazole moiety in the minor groove leaving the β-carboline chromophore outside. The estimated binding energy values for these two interaction forms are −16 and −186 kcal/mole, respectively. In these experiments, the occurrence of hydrogen bonding was observed in both conformations and resulted in the formation of a stable drug-DNA complex. The hydrogen bonding between the N-9 of β-carboline and the oxygen atom of C-2 in thymidine had a bonding length 1.86 and 2.02 Ǻ, respectively [22].

## 3. Experimental Section

### 3.1. General Experimental Procedures

All of melting points were taken on a Buchi mp B-540 apparatus and were uncorrected. UV and IR spectra were recorded on Hitachi U-3210 and JASCO A-100 IR spectrophotometers, respectively. EIMS spectra were obtained on a MAT 112S-JMS D300 spectrometer, using direct inlet systems. HRMS data were taken on a JMX 110 mass spectrometer. ^1^H- and ^13^C-NMR spectra were recorded on a Bruker FT-300 spectrometer. Analytical thin-layer chromatography (TLC) was carried out on Kiesel gel GF_254_ plates and detection was made under UV light. EM Kieselgel 60 (230-400 mesh ASTM) was used for column chromatography.

### 3.2. Synthesis of Compounds **1**, **3**, **5**, **7**, **9**, **11**, **13**, **15**, and **17**

To a stirred solution of tryptamine (160 mg, 0.1 mmol), the appropriate substituted aldehyde (**27**-**34**, and *N*-ethyl-3-carbazolyl carboxyaldehyde; 0.1 mmol) in acetic acid (30 mL) was heated to 100 °C. The reaction mixture was maintained at 100 °C for 24 h. After cooling, the reaction mixture was neutralized with NH_4_OH solution and extracted with CHCl_3_. The CHCl_3_ layer was evaporated under vacuum, and the residue was chromatographed on a Silica gel column (10 g) and eluted with solvent mixture of CHCl_3_/MeOH using the following ratios and volumes (70:1, 50:1, 30:1 and 10:1; each 30 mL), to afford compounds **1**, **3**, **5**, **7**, **9**, **11**, **13**, **15**, and **17** with a yield in the range of 80-85%.

3.2.1. 1-(9′-Ethyl-3′-carbazolyl)-1,2,3,4-tetrahydro-β-carboline (**1**) 

Yellow solid; mp 240-242 °C; UV λ_max_ 249, 269, 296, 332, 346 nm; IR (KBr) ν_max_ 3164, 2973, 1600, 1471, 1334, 806 cm^−1^; ^1^H NMR (CDCl_3_) δ_Η_ 5.36 (1H, s, H-1), 3.37 (1H, m, H-3a), 3.13 (1H, m, H-3b), 2.87 (1H, m, H-4a), 2.97 (1H, m, H-4b), 7.64 (1H, d, overlap, H-5), 7.17 (1H, m, H-6), 7.14 (1H, m, H-7), 7.11 (1H, d, overlap, H-8), 7.29 (1H, d, overlap, H-1′), 7.36 (1H, d, overlap, H-2′), 8.04 (1H, s, H-4′), 7.44 (1H, d, overlap, H-5′), 7.52 (1H, m, H-6′), 7.25 (1H, m, H-7′), 8.00 (1H, d, overlap, H-8′), 4.37 (2H, q, *J* = 7.1 Hz, H-1″), 1.45 (3H, t, *J* = 7.1 Hz, H-2″); ^13^C NMR (CDCl_3_) δ_C_ 58.4 (d, C-1), 43.0 (t, C-3), 43.0 (t, C-4), 110.0 (s, C-4a), 127.6 (s, C-4b), 118.2 (d, C-5), 119.3 (d, C-6), 121.6 (d, C-7), 111.1 (d, C-8), 136.1 (s, C-8a), 135.5 (s, C-9a), 108.7 (d, C-1′), 126.5 (d, C-2′), 132.3 (s, C-3′), 120.7 (d, C-4′), 122.8 (s, C-4′a), 123.1 (s, C-4′b), 108.7 (d, C-5′), 126.0 (d, C-6′), 119.1 (d, C-7′), 120.5 (d, C-8′), 139.8 (s, C-8′a), 140.4 (s, C-9′a), 37.6 (t, C-1″), 13.9 (q, C-2″); EIMS *m*/*z* 365 (100, M^+^), 364 (82), 336 (46), 335 (63), 306 (31), 183 (17), 171 (51), 160 (16), 143 (18), 115 (17), 77 (5); HREIMS *m*/*z* 365.1890 ([M]^+^, calcd. for C_25_H_23_N_3_, 365.1892). 

3.2.2. 1-(9′-Propyl-3′-carbazolyl)-1,2,3,4-tetrahydro-β-carboline (**3**) 

Yellow solid; mp 110-112 °C; UV λ_max_ 269, 296, 332, 346 nm; IR (KBr) ν_max_ 3168, 3050, 2964, 1598, 1469, 1371, 808 cm^−1^; ^1^H NMR (CDCl_3_) δ_Η_ 5.37 (1H, s, H-1), 3.18 (1H, m, H-3a), 3.43 (1H, m, H-3b), 2.85 (1H, m, H-4a), 2.99 (1H, m, H-4b), 7.58 (1H, d, overlap, H-5), 7.19 (1H, m, H-6), 7.17 (1H, m, H-7), 7.16 (1H, d, overlap, H-8), 7.34 (1H, d, overlap, H-1′), 7.41 (1H, d, overlap, H-2′), 8.02 (1H, s, H-4′), 7.43 (1H, d, overlap, H-5′), 7.48 (1H, dd, overlap, H-6′), 7.23 (1H, dd, overlap, H-7′), 7.98 (1H, d, overlap, H-8′), 4.24 (2H, t, *J* = 7.0 Hz, H-1″), 1.89 (2H, m, H-2″), 0.96 (3H, t, *J* = 7.3 Hz, H-3″);^13^C NMR (CDCl_3_) δ_C_58.3 (d, C-1), 42.8 (t, C-3), 22.4 (t, C-4), 109.9 (s, C-4a), 127.5 (s, C-4b), 118.2 (d, C-5), 119.3 (d, C-6), 121.6 (d, C-7), 111.1 (d, C-8), 136.1 (s, C-8a), 135.0 (s, C-9a), 109.7 (d, C-1′), 126.4 (d, C-2′), 131.7 (s, C-3′), 120.6 (d, C-4′), 122.6 (s, C-4′a), 122.9 (s, C-4′b), 108.9 (d, C-5′), 125.9 (d, C-6′), 119.1 (d, C-7′), 120.4 (d, C-8′), 140.4 (s, C-8′a), 140.9 (s, C-9′a), 44.7 (t, C-1″), 22.4 (t, C-2″)11.8 (q, C-3″); EIMS *m*/*z* 379 (89, M^+^), 378 (100, M-1^+^),350 (65), 306 (31), 190 (22), 180 (22), 171 (58), 159 (43), 115 (13), 77 (4). 

3.2.3. 1-(9′-[1″-Methyl]propyl-3′-carbazolyl)-1,2,3,4-tetrahydro-β-carboline (**5**) 

Yellow solid; mp 127-130 °C; UV λ_max_ 222, 297, 332, 346 nm; IR (KBr) ν_max_ 3454, 2969, 2929, 1458, 1335, 742 cm^−1^; ^1^H NMR (CDCl_3_) δ_H_ 5.31 (1H, s, H-1), 3.16 (1H, m, H-3a), 3.42 (1H, m, H-3b), 2.87 (1H, m, H-4a), 2.97 (1H, m, H-4b), 7.64 (1H, d, overlap, H-5), 7.15 (1H, m, H-6), 7.15 (1H, m, H-7), 7.15 (1H, d, overlap, H-8), 7.53 (1H, d, overlap, H-1′), 7.33(1H, d, overlap, H-2′), 8.04 (1H, s, H-4′), 7.45 (1H, d, overlap, H-5′), 7.45 (1H, dd, overlap, H-6′), 7.21 (1H, dd, overlap, H-7′), 8.02 (1H, d, overlap, H-8′), 4.67 (2H, m, H-1″), 1.68 (2H, d, *J* = 6.9 Hz, H-2″), 2.03 (1H, m, H-3a″), 2.15 (1H, m, H-3b″), 0.96 (3H, t, *J* = 7.3 Hz, H-4″); ^13^C NMR (CDCl_3_) δ_C_ 58.3 (d, C-1), 43.0 (t, C-3), 19.1 (t, C-4), 110.0 (s, C-4a), 127.5 (s, C-4b), 118.2 (d, C-5), 109.3 (d, C-6), 121.5 (d, C-7), 110.1 (d, C-8), 135.9 (s, C-8a), 135.1 (s, C-9a), 110.3 (d, C-1′), 126.0 (d, C-2′), 131.6 (s, C-3′), 120.5 (d, C-4′), 122.9 (s, C-4′a), 123.8 (s, C-4′b), 110.2 (d, C-5′), 125.6 (d, C-6′), 118.7 (d, C-7′), 120.3 (d, C-8′), 140.5 (s, C-8′a), 139.7 (s, C-9′a), 53.0 (t, C-1″), 14.1 (t, C-2″), 28.0 (t, C-3″), 11.5 (t, C-4″); EIMS *m*/*z* 393 (100, M^+^), 364 (36), 336 (17), 306 (34), 193 (20), 180 (11), 171 (44), 144 (20), 115 (19), 57 (42). 

3.2.4. 1-(9′-Pentyl-3′-carbazolyl)-1,2,3,4-tetrahydro-β-carboline (**7**) 

Yellow solid; mp 129-130 °C; UV λ_max_ 243, 266, 296, 332, 347 nm; IR (KBr) ν_max_ 3405, 3164, 3050, 2929, 1600, 1467, 1338, 806 cm^−1^; ^1^H NMR (CDCl_3_) δ_H_ 5.28 (1H, s, H-1), 3.14 (1H, m, H-3a), 3.41 (1H, m, H-3b), 2.85 (1H, m, H-4a), 2.99 (1H, m, H-4b), 7.63 (1H, d, overlap, H-5), 7.19 (1H, m, H-6), 7.18 (1H, m, H-7), 7.16 (1H, d, overlap, H-8), 7.34 (1H, d, overlap, H-1′), 7.37 (1H, d, overlap, H-2′), 8.03 (1H, s, H-4′), 7.42 (1H, d, overlap, H-5′), 7.49 (1H, dd, overlap, H-6′), 7.25 (1H, dd, overlap, H-7′), 7.99 (1H, d, overlap, H-8′), 4.27 (2H, t, *J* = 7.1 Hz, H-1″), 1.91 (2H, m, H-2″), 1.41 (2H, m, H-3″), 1.41 (2H, m, H-4″), 0.96 (3H, t, *J* = 7.3 Hz, H-2″); ^13^C NMR (CDCl_3_) δ_C_ 58.4 (d, C-1), 43.1 (t, C-3), 22.6 (t, C-4), 110.0 (s, C-4a), 127.6 (s, C-4b), 118.2 (d, C-5), 119.3 (d, C-6), 121.6 (d, C-7), 111.0 (d, C-8), 136.0 (s, C-8a), 135.4 (s, C-9a), 109.0 (d, C-1′), 126.4 (d, C-2′), 132.1 (s, C-3′), 120.6 (d, C-4′), 122.6 (s, C-4′a), 123.0 (s, C-4′b), 108.9 (d, C-5′), 125.9 (d, C-6′), 119.0 (d, C-7′), 120.4 (d, C-8′), 140.3 (s, C-8′a), 140.9 (s, C-9′a), 43.2 (t, C-1″), 28.7 (t, C-2″), 29.4 (t, C-3″), 22.5 (t, C-4″), 14.0 (q, C-5″); EIMS *m*/*z* 407 (90, M^+^), 406 (100, M − H^+^), 378 (55), 306 (31), 204 (13), 179 (19), 171 (46), 159 (27), 115 (15), 55 (8). 

3.2.5. 1-(9′-[3″-Methylbutyl]-3′-carbazolyl)-1,2,3,4-tetrahydro-β-carboline (**9**) 

Yellow solid; mp 99-101 °C; UV λ_max_ 222, 296 nm; IR (KBr) ν_max_ 3404, 3192, 3050, 2922, 1468, 1331, 742 cm^−1^; ^1^H NMR (CDCl_3_) δ_H_ 5.34 (1H, s, H-1), 3.18 (1H, m, H-3a), 3.46 (1H, m, H-3b), 2.87 (1H, m, H-4a), 3.00 (1H, m, H-4b), 7.64 (1H, d, overlap, H-5), 7.17 (1H, dd, overlap, H-6), 7.15 (1H, dd, overlap, H-7), 7.14 (1H, d, overlap, H-8), 7.36 (1H, d, overlap, H-1′), 7.40 (1H, d, overlap, H-2′), 8.04 (1H, s, H-4′), 7.47 (1H, d, overlap, H-5′), 7.56 (1H, dd, overlap, H-6′), 7.22 (1H, dd, overlap, H-7′), 8.01 (1H, d, overlap, H-8′), 4.29 (2H, t, *J* = 7.2 Hz, H-1″), 1.73 (2H, m, H-2″), 1.73 (2H, m, H-3″), 1.02 (2H, d, *J* = 6.0 Hz, H-4″), 1.02 (3H, d, *J* = 6.0 Hz, H-5″); ^13^C NMR (CDCl3) δ_C_ 58.1 (d, C-1), 42.6 (t, C-3), 22.3 (t, C-4), 109.7 (s, C-4a), 127.3 (s, C-4b), 117.9 (d, C-5), 119.0 (d, C-6), 121.3 (d, C-7), 110.8 (d, C-8), 135.8 (s, C-8a), 135.2 (s, C-9a), 108.1 (d, C-1′), 126.2 (d, C-2′), 132.0 (s, C-3′), 120.4 (d, C-4′), 122.5 (s, C-4′a), 122.8 (s, C-4′b), 108.6 (d, C-5′), 125.7 (d, C-6′), 118.8 (d, C-7′), 120.2 (d, C-8′), 140.5 (s, C-8′a), 139.9 (s, C-9′a), 41.2 (t, C-1″), 37.3 (t, C-2″), 25.9 (t, C-3″), 22.4 (t, C-4″), 22.4 (q, C-5″); EIMS *m*/*z* 407 (90, M^+^), 378 (48), 319 (15), 306 (25), 204 (29), 180 (23), 171 (64), 160 (51), 55 (81), 43 (57). 

3.2.6. 1-(9′-Octyl-3′-carbazolyl)-1,2,3,4-tetrahydro-β-carboline (**11**) 

Yellow solid; mp 87-89 °C; UV λ_max_ 247, 266, 296, 332, 347 nm; IR (KBr) ν_max_ 3160, 3056, 2927, 1600, 1469, 1332, 806, 742 cm^−1^; ^1^H NMR (CDCl_3_) δ_H_ 5.32 (1H, s, H-1), 3.18 (1H, m, H-3a), 3.44 (1H, m, H-3b), 2.88 (1H, m, H-4a), 2.97 (1H, m, H-4b), 7.64 (1H, d, overlap, H-5), 7.19 (1H, m, H-6), 7.17 (1H, m, H-7), 7.15 (1H, d, overlap, H-8), 7.33 (1H, d, overlap, H-1′), 7.39 (1H, d, overlap, H-2′), 8.04 (1H, s, H-4′), 7.47 (1H, d, overlap, H-5′), 7.54 (1H, dd, overlap, H-6′), 7.27 (1H, dd, overlap, H-7′), 8.01 (1H, d, overlap, H-8′), 4.27 (2H, t, *J* = 7.0 Hz, H-1″), 1.86 (2H, m, H-2″), 1.26-1.35 (10H, overlap, H-3″~H-7″), 0.88 (3H, t, *J* = 6.7 Hz, H-8″); ^13^C NMR (CDCl_3_) δ_C_ 58.4 (d, C-1), 42.9 (t, C-3), 22.5 (t, C-4), 110.0 (s, C-4a), 127.6 (s, C-4b), 118.2 (d, C-5), 119.1 (d, C-6), 121.6 (d, C-7), 111.1 (d, C-8), 136.1 (s, C-8a), 135.3 (s, C-9a), 109.0 (d, C-1′), 126.4 (d, C-2′), 132.1 (s, C-3′), 120.6 (d, C-4′), 122.6 (s, C-4′a), 123.0 (s, C-4′b), 108.9 (d, C-5′), 125.9 (d, C-6′), 118.7 (d, C-7′), 120.4 (d, C-8′), 140.3 (s, C-8′a), 140.9 (s, C-9′a), 43.2 (t, C-1″), 29.1 (t, C-2″), 27.4 (t, C-3″), 29.5 (t, C-4″), 29.3 (t, C-5″), 31.8 (t, C-6″), 22.7 (t, C-7″), 14.2 (q, C-8″); EIMS *m*/*z* 449 (83, M^+^), 448 (100, M − H^+^), 420 (57), 405 (7), 319 (20), 306 (25), 225 (9), 180 (17), 171 (37), 160 (15), 57 (11). 

3.2.7. 1-(9′-Decyl-3′-carbazolyl)-1,2,3,4-tetrahydro-β-carboline (**13**) 

Yellow solid; mp 102-104 °C; UV λ_max_ 240, 266, 296, 332, 347 nm; IR (KBr) *v*_max_ 3048, 2925, 1598, 1467, 1332, 806 cm^−1^; ^1^H NMR (CDCl_3_) δ_H_ 5.37 (1H, s, H-1), 3.18 (1H, m, H-3a), 3.43 (1H, m, H-3b), 2.88 (1H, m, H-4a), 2.99 (1H, m, H-4b), 7.55 (1H, d, overlap, H-5), 7.15 (1H, m, H-6), 7.14 (1H, dd, m), 7.16 (1H, d, overlap, H-8), 7.33 (1H, d, overlap, H-1′), 7.40 (1H, d, overlap, H-2′), 8.02 (1H, s, H-4′), 7.38 (1H, d, overlap, H-5′), 7.47 (1H, dd, overlap, H-6′), 7.20 (1H, dd, overlap, H-7′), 8.00 (1H, d, overlap, H-8′), 4.25 (2H, t, *J* = 7.0 Hz, H-1″), 1.83 (2H, m, H-2″), 1.24-1.32 (14H, overlap, H-3″~H-9″), 0.88 (3H, t, *J* =6.3 Hz, H-10″); ^13^C NMR (CDCl_3_) δ_C_ 58.0 (d, C-1), 42.4 (t, C-3), 21.6 (t, C-4), 109.8 (s, C-4a), 127.2 (s, C-4b), 118.2 (d, C-5), 119.4 (d, C-6), 121.8 (d, C-7), 110.9 (d, C-8), 135.9 (s, C-8a), 133.7 (s, C-9a), 108.9 (d, C-1′), 126.4 (d, C-2′), 130.1 (s, C-3′), 120.7 (d, C-4′), 122.4 (s, C-4′a), 122.9 (s, C-4′b), 108.7 (d, C-5′), 125.9 (d, C-6′), 119.0 (d, C-7′), 120.6 (d, C-8′), 140.4 (s, C-8′a), 140.7 (s, C-9′a), 43.1 (t, C-1″), 28.9 (t, C-2″), 27.2 (t, C-3″), 29.2-29.5 (t, C-4″~C-7″), 31.8 (t, C-5″), 31.8 (t, C-8″), 22.6 (t, C-9″), 14.0 (q, C-10″); EIMS *m*/*z* 477 (87, M^+^), 476 (100, M − H^+^), 448 (46), 306 (21), 239 (15), 180 (25), 171 (56), 160 (43), 144 (13), 69 (12), 57 (36). 

3.2.8. 1-(9′-Hexadecyl-3′-carbazolyl)-1,2,3,4-tetrahydro-β-carboline (**15**) 

Yellow solid; mp 111-113 °C; UV λ_max_ 240, 270 nm; IR (KBr) ν_max_ 2952, 2923, 1467 cm^−^^1^; ^1^H NMR (CDCl_3_) δ_H_ 5.34 (1H, s, H-1), 3.15 (1H, m, H-3a), 3.39 (1H, m, H-3b), 2.86 (1H, m, H-4a), 3.00 (1H, m, H-4b), 7.60 (1H, d, overlap, H-5), 7.14 (1H, dd, overlap, H-6), 7.16 (1H, dd, overlap, m), 7.17 (1H, d, overlap, H-8), 7.36 (1H, d, overlap, H-1′), 7.39 (1H, d, overlap, H-2′), 8.01 (1H, s, H-4′), 7.34 (1H, d, overlap, H-5′), 7.48 (1H, dd, overlap, H-6′), 7.21 (1H, dd, overlap, H-7′), 8.03 (1H, d, overlap, H-8′), 4.24 (2H, t, *J* = 7.2 Hz, H-1″), 1.83 (2H, m, H-2″), 1.26-1.33 (14H, overlap, H-3″~H-15″), 0.90 (3H, t, *J* = 6.6 Hz, H-16″); ^13^C NMR (CDCl_3_) δ_C_ 58.4 (d, C-1), 43.2 (t, C-3), 22.7 (t, C-4), 109.9 (s, C-4a), 127.4 (s, C-4b), 118.2 (d, C-5), 119.4 (d, C-6), 121.7 (d, C-7), 110.9 (d, C-8), 134.9 (s, C-8a), 135.9 (s, C-9a), 109.0 (d, C-1′), 126.3 (d, C-2′), 131.3 (s, C-3′), 120.4 (d, C-4′), 122.5 (s, C-4′a), 122.9 (s, C-4′b), 108.8 (d, C-5′), 125.8 (d, C-6′), 119.0 (d, C-7′), 120.6 (d, C-8′), 140.8 (s, C-8′a), 140.4 (s, C-9′a), 43.2 (t, C-1″), 22.7-31.9 (t, C-4″~C-15″), 14.1 (q, C-16″) ; EIMS *m*/*z* 561 (4, M^+^), 368 (1), 313 (1), 236 (3), 180 (2), 97 (6), 83 (11), 69 (17), 57 (28), 44 (100). 

3.2.9. 1-(9′-Eicosyl-3′-carbazolyl)-1,2,3,4-tetrahydro-β-carboline (**17**) 

Yellow amorphous powder; UV λ_max_ 221, 263 nm; IR (KBr) ν_max_ 3048, 2925, 1598, 1467, 1332, 806 cm^−^^1^; ^1^H NMR (CDCl_3_) δ_H_ 5.38 (1H, s, H-1), 3.18 (1H, m, H-3a), 3.41 (1H, m, H-3b), 2.88 (1H, m, H-4a), 3.01 (1H, m, H-4b), 7.59 (1H, d, overlap, H-5), 7.14 (1H, dd, overlap, H-6), 7.16 (1H, dd, overlap, m), 7.17 (1H, d, overlap, H-8), 7.36 (1H, d, overlap, H-1′), 7.39 (1H, d, overlap, H-2′), 8.01 (1H, s, H-4′), 7.34 (1H, d, overlap, H-5′), 7.46 (1H, dd, overlap, H-6′), 7.21 (1H, dd, overlap, H-7′), 8.03 (1H, d, overlap, H-8′), 4.24 (2H, t, *J* = 7.2 Hz, H-1″), 1.83 (2H, m, H-2″), 1.26-1.33 (14H, overlap, H-3″~H-19″), 0.91 (3H, t, *J* = 6.7 Hz, H-20″); ^13^C NMR (CDCl_3_) δ_C_ 57.2 (d, C-1), 40.8 (t, C-3), 20.0 (t, C-4), 108.5 (s, C-4a), 127.3 (s, C-4b), 117.9 (d, C-5), 119.1 (d, C-6), 121.9 (d, C-7), 111.0 (d, C-8), 130.8 (s, C-8a), 136.2 (s, C-9a), 108.8 (d, C-1′), 126.3 (d, C-2′), 131.8 (s, C-3′), 120.2 (d, C-4′), 122.1 (s, C-4′a), 122.8 (s, C-4′b), 108.7 (d, C-5′), 125.8 (d, C-6′), 118.9 (d, C-7′), 120.8 (d, C-8′), 140.6 (s, C-8′a), 140.5 (s, C-9′a), 42.9 (t, C-1″), 22.4-31.6 (t, C-2″~C-19″), 13.7 (q, C-20″); EIMS *m*/*z* 617 (4, M^+^), 588 (1), 179 (6), 171 (26), 160 (43), 144 (4), 91 (4), 71 (15), 69 (10), 57 (59), 43 (100).

### 3.3. Synthesis of Compounds **2**, **4**, **6**, **8**, **10**, **12**, **14**, **16**, and **18**

To a stirred solution of compound A (**1**, **3**, **5**, **7**, **9**, **11**, **13**, **15**, **17**, 0.68 mmol) in EtOH (2 mL) and CHCl_3_ (6 mL) at room temperature, 2,3-dichloro-5,6-dicyanobezoquinone (DDQ, 160 mg) was added. The reaction mixture was stirred for 30 mins. After concentration, the residue was applied on a prep. TLC and developed with CHCl_3_/MeOH (10:1) to yield a series of B (**2**, **4**, **6**, **8**, **10**, **12**, **14**, **16**, and **18**) (23-64% yield). 

3.3.1. 1-(9′-Ethyl-3′-carbazolyl)-3,4-dihydro-β-carboline (**2**) 

Yellow solid; mp 175-177 °C; UV λ_max_ 236, 288, 323, 346 nm; IR (KBr) ν_max_ 3164, 2973, 1600, 1471, 1334, 806 cm^−1^; ^1^H NMR (CDCl_3_) δ_H_ 3.70 (2H, t, *J* = 7.5 Hz, H-3), 2.93 (2H, t, *J* = 7.5 Hz, H-4), 7.64 (1H, d, overlap, H-5), 7.19 (1H, m, H-6), 7.25 (1H, m, H-7), 7.43 (1H, d, overlap, H-8), 7.32 (1H, d, overlap,H-1′), 7.87 (1H, d, overlap, H-2′), 8.52 (1H, s, H-4′), 7.30 (1H, d, overlap, H-5′), 7.46 (1H, m, H-6′), 7.22 (1H, m, H-7′), 8.01 (1H, d, overlap, H-8′), 4.14 (2H, q, *J* = 7.0 Hz, H-1″), 1.34 (3H, t, *J* = 7.0 Hz, H-2″); ^13^C NMR (CDCl_3_) δ_C_ 160.5 (s, C-1), 46.4 (t, C-3), 19.4 (t, C-4), 112.8 (s, C-4a), 127.7 (s, C-4b), 120.1 (d, C-5), 120.6 (d, C-6), 125.2 (d, C-7), 112.8 (d, C-8), 138.1 (s, C-8a), 127.7 (s, C-9a), 108.7 (d, C-1′), 126.8 (d, C-2′), 141.4 (s, C-3′), 121.4 (d, C-4′), 122.9 (s, C-4′a), 123.1 (s, C-4′b), 108.5 (d, C-5′), 126.1 (d, C-6′), 119.5 (d, C-7′), 121.1 (d, C-8′), 126.1 (s, C-8′a), 140.3 (s, C-9′a), 37.4 (t, C-1″), 13.7 (q, C-2″); EIMS *m*/*z* 363 (100, M^+^), 362 (90), 346 (16), 335 (20), 319 (9), 306 (10), 174 (15), 160 (19), 77 (4), 55 (11); HREIMS *m*/*z* 363.1724 ([M]^+^, calcd. for C_25_H_21_N_3_, 363.1735). 

3.3.2. 1-(9′-Propyl-3′-carbazolyl)-3,4-dihydro-β-carboline (**4**) 

Yellow solid; mp 126-128 °C; UV λ_max_ 239, 288, 323 nm; IR (KBr) ν_max_ 3064, 2929, 1596, 1467, 1336, 808, 746 cm^−1^; ^1^H NMR (CDCl_3_) δ_H_ 3.96 (2H, t, *J* = 8.2 Hz, H-3), 2.98 (2H, t, *J* = 8.3 Hz, H-4), 7.64 (1H, d, overlap, H-5), 7.17 (1H, m, H-6), 7.26 (1H, m, H-7), 7.44 (1H, d, overlap, H-8), 7.38 (1H, d, overlap, H-1′), 7.92 (1H, d, overlap, H-2′), 8.58 (1H, s, H-4′), 7.34 (1H, d, overlap, H-5′), 7.47 (1H, m, H-6′), 7.21 (1H, m, H-7′), 8.09 (1H, d, 7.6, H-8′), 4.21 (2H, t, *J* = 6.9 Hz, H-1″), 1.88 (2H, m, H-2″), 0.95(3H, t, 7.4); ^13^C NMR (CDCl_3_) δ_H_ 160.2 (s, C-1), 47.3 (t, C-3), 19.4 (t, C-4), 119.0 (s, C-4a), 125.4 (s, C-4b), 120.1 (d, C-5), 120.5 (d, C-6), 125.0 (d, C-7), 112.5 (d, C-8), 137.5 (s, C-8a), 127.8 (s, C-9a), 109.1 (d, C-1′), 126.5 (d, C-2′), 141.9 (s, C-3′), 120.9 (d, C-4′), 122.8 (s, C-4′a), 123.0 (s, C-4′b), 109.0 (d, C-5′), 126.3 (d, C-6′), 119.5 (d, C-7′), 120.8 (d, C-8′), 126.2 (s, C-8′a), 141.0 (s, C-9′a), 44.7 (t, C-1″), 22.3(t, C-2″), 11.7 (q, C-3″); EIMS *m*/*z* 377 (98, M^+^), 376 (100, M − H^+^), 346 (55), 319 (15), 306 (15), 205 (14), 180 (8), 174 (47), 159 (16), 115 (11), 77 (9), 57 (21); HREIMS *m*/*z* 377.1880 ([M]^+^, calcd. for C_26_H_23_N_3_, 377.1882). 

3.3.3. 1-(9′-[1″-Methyl]propyl-3′-carbazolyl)-3,4-dihydro-β-carboline (**6**) 

Yellow solid; mp 151-154 °C; UV λ_max_ 237, 288 nm; IR (KBr) ν_max_ 3051, 2921, 1468, 1335, 743 cm^−1^; ^1^H NMR (CDCl^3^) δ_H_ 3.67 (2H, t, *J* = 8.1 Hz, H-3), 2.88 (2H, t, *J* = 8.1 Hz, H-4), 7.68 (1H, d, overlap, H-5), 7.19 (1H, m, H-6), 7.37 (1H, m, H-7), 7.56 (1H, d, overlap, H-8), 7.43 (1H, d, overlap,H-1′), 8.05 (1H, d, overlap, H-2′), 8.83 (1H, s, H-4′), 7.45 (1H, d, overlap, H-5′), 7.34 (1H, m, H-6′), 7.08 (1H, m, H-7′), 8.08 (1H, d, 7.6, H-8′), 4.56 (2H, t, *J* = 6.9 Hz, H-1″), 1.56 (2H, m, H-2″), 1.95 (3H, m, H-3″), 0.70 (3H, m, H-4″); ^13^C NMR (CDCl_3_) δ_C_ 160.2 (s, C-1), 43.0 (t, C-3), 19.4 (t, C-4), 113.9 (s, C-4a), 125.8 (s, C-4b), 120.9 (d, C-5), 121.6 (d, C-6), 126.8 (d, C-7), 113.9 (d, C-8), 140.9 (s, C-8a), 126.2 (s, C-9a), 111.0 (d, C-1′), 127.9 (d, C-2′), 140.8 (s, C-3′), 123.7 (d, C-4′), 123.2 (s, C-4′a), 123.7 (s, C-4′b), 111.0 (d, C-5′), 127.9 (d, C-6′), 120.5 (d, C-7′), 121.8 (d, C-8′), 124.0 (s, C-8′a), 140.1 (s, C-9′a), 53.7 (t, C-1″), 19.1 (q, C-2″), 28.1 (t, C-3″), 11.6 (q, C-4″); EIMS *m*/*z* 391 (72, M^+^), 360 (100), 332 (45), 306 (33), 180 (74), 167 (40), 140 (20), 115 (31), 57 (35), 41 (88); HRESIMS *m*/*z* 392.2125 ([M + H]^+^, calcd. for C_27_H_26_N_3_, 392.2127).

3.3.4. 1-(9′-Pentyl-3′-carbazolyl)-3,4-dihydro-β-carboline (**8**) 

Yellow solid; mp 97-98 °C; UV λ_max_ 236, 288, 324 nm; IR (KBr) ν_max_ 3064, 2929, 1601, 1467, 1334, 808 cm^−1^; ^1^H NMR (CDCl_3_) δ_H_ 3.40 (2H, t, *J* = 7.0 Hz, H-3), 2.91 (2H, t, *J* = 7.8 Hz, H-4), 7.62 (1H, d, overlap, H-5), 7.21 (1H, m, H-6), 7.34 (1H, m, H-7), 7.57 (1H, d, overlap, H-8), 7.27 (1H, d, overlap,H-1′), 7.97 (1H, d, overlap, H-2′), 8.64 (1H, s, H-4′), 7.31 (1H, d, overlap, H-5′), 7.48 (1H, m, H-6′), 7.25 (1H, m, H-7′), 8.06 (1H, d, 7.6, H-8′), 3.90 (2H, t, *J* = 6.9 Hz, H-1″), 1.71 (2H, m, H-2″), 1.28 (3H, m, H-3″), 1.34 (3H, m, H-4″), 0.84 (t, 6.6, H-5″); ^13^C NMR (CDCl_3_) δ_C_ 160.5 (s, C-1), 44.9 (t, C-3), 19.3 (t, C-4), 119.4 (s, C-4a), 124.2 (s, C-4b), 120.1 (d, C-5), 120.6 (d, C-6), 125.7 (d, C-7), 113.0 (d, C-8), 138.8 (s, C-8a), 127.2 (s, C-9a), 108.7 (d, C-1′), 127.0 (d, C-2′), 142.0 (s, C-3′), 121.7 (d, C-4′), 122.6 (s, C-4′a), 123.0 (s, C-4′b), 108.6 (d, C-5′), 126.0 (d, C-6′), 119.4 (d, C-7′), 121.1 (d, C-8′), 124.8 (s, C-8′a), 140.5 (s, C-9′a), 42.6 (t, C-1″), 28.3 (t, C-2″), 29.1 (t, C-3″), 22.2 (t, C-4″), 13.8 (q, C-5″); EIMS *m*/*z* 405 (83, M^+^), 404 (100, M − H^+^), 377 (25), 346 (29), 332 (9), 306 (13), 203 (10), 174 (55), 159 (25), 57 (16); HREIMS *m*/*z* 405.2199 ([M]^+^, calcd. for C_28_H_27_N_3_, 405.2205). 

3.3.5. 1-(9′-[3″-Methylbutyl]-3′-carbazolyl)-3,4-dihydro-β-carboline (**10**) 

Yellow solid; mp 135-137 °C; UV λ_max_ 236, 289 nm; IR (KBr) ν_max_ 3068, 2927, 1587, 1465, 1332, 741 cm^−^^1^; ^1^H NMR (CDCl_3_) δ_H_ 3.56 (2H, t, *J* = 8.0 Hz, H-3), 2.76 (2H, t, *J* = 8.0 Hz, H-4), 7.41 (1H, d, overlap, H-5), 7.16 (1H, m, H-6), 7.41 (1H, m, H-7), 7.79 (1H, d, overlap, H-8), 7.24 (1H, d, overlap,H-1′), 8.03 (1H, d, overlap, H-2′), 8.86 (1H, s, H-4′), 7.16 (1H, d, overlap, H-5′), 7.36 (1H, m, H-6′), 7.14 (1H, m, H-7′), 8.12 (1H, d, *J* = 7.6 Hz, H-8′), 3.74 (2H, t, *J* = 6.9 Hz, H-1″), 1.44 (2H, m, H-2″), 1.54 (2H, m, H-3″), 0.90 (6H, d, *J* = 7.6 Hz, H-4″, 5″); ^13^C NMR (CDCl_3_) δ_C_ 160.9 (s, C-1), 41.2 (t, C-3), 19.3 (t, C-4), 114.3 (s, C-4a), 126.8 (s, C-4b), 120.6 (d, C-5), 121.5 (d, C-6), 126.8 (d, C-7), 114.3 (d, C-8), 140.6 (s, C-8a), 126.8 (s, C-9a), 109.3 (d, C-1′), 128.8 (d, C-2′), 143.1 (s, C-3′), 123.4 (d, C-4′), 121.7 (s, C-4′a), 122.5 (s, C-4′b), 108.9 (d, C-5′), 128.0 (d, C-6′), 120.4 (d, C-7′), 121.6 (d, C-8′), 124.2 (s, C-8′a), 140.6 (s, C-9′a), 41.2 (t, C-1″), 37.1 (q, C-2″), 26.1 (t, C-3″), 22.4 (q, C-4″, 5″); EIMS *m*/*z* 405 (22, M^+^), 346 (13), 332 (3), 180 (2), 174 (10), 160 (5), 83 (7), 69 (12), 55 (20), 44 (100); HRESIMS *m*/*z* 406.2284 ([M + H]^+^, calcd. for C_28_H_28_N_3_, 406.2283).

3.3.6. 1-(9′-Octyl-3′-carbazolyl)-3,4-dihydro-β-carboline (**12**) 

Yellow solid; mp 91-92 °C; UV λ_max_ 226, 287, 324 nm; IR (KBr) ν_max_ 3058, 2927, 1596, 1467, 1336, 744 cm^−1^; ^1^H NMR (CDCl_3_) δ_H_ 3.77 (2H, t, *J* = 8.3 Hz, H-3), 2.88 (2H, t, *J* = 8.7 Hz, H-4), 7.62 (1H, d, overlap, H-5), 7.17 (1H, m, H-6), 7.30 (1H, m, H-7), 7.45 (1H, d, overlap, H-8), 7.33 (1H, d, overlap,H-1′), 7.87 (1H, d, overlap, H-2′), 8.53 (1H, s, H-4′), 7.36 (1H, d, overlap, H-5′), 7.51 (1H, m, H-6′), 7.19 (1H, m, H-7′), 8.03 (1H, d, 7.6, H-8′), 4.08 (2H, t, *J* = 6.9 Hz, H-1″), 1.76 (2H, m, H-2″), 1.30 (3H, m, H-3″), 1.27-1.29 (8H, m, H-4″~H-7″), 0.92 (t, 6.1, H-8″); ^13^C NMR (CDCl_3_) δ_C_ 160.1 (s, C-1), 44.8 (t, C-3), 19.4 (t, C-4), 118.1 (s, C-4a), 125.6 (s, C-4b), 120.0 (d, C-5), 120.3 (d, C-6), 124.5 (d, C-7), 112.2 (d, C-8), 136.8 (s, C-8a), 128.3 (s, C-9a), 109.0 (d, C-1′), 126.5 (d, C-2′), 141.5 (s, C-3′), 120.7 (d, C-4′), 122.9 (s, C-4′a), 122.9 (s, C-4′b), 108.9 (d, C-5′), 125.8 (d, C-6′), 119.3 (d, C-7′), 120.4 (d, C-8′), 128.0 (s, C-8′a), 141.0 (s, C-9′a), 43.2 (t, C-1″), 29.0 (t, C-2″), 27.3 (t, C-3″), 29.4 (t, C-4″), 29.2 (t, C-5″), 31.8 (t, C-6″), 22.6 (t, C-7″), 14.2 (q, C-8″); EIMS *m*/*z* 447 (81, M^+^), 446 (100, M − H^+^), 432 (6), 419 (20), 404 (8), 362 (10), 346 (29), 319 (15), 174 (67), 160 (9), 69 (23), 57 (46); HREIMS *m*/*z* 447.2673 ([M]^+^, calcd. for C_31_H_33_N_3_, 447.2674). 

3.3.7. 1-(9′-Decyl-3′-carbazolyl)-3,4-dihydro-β-carboline (**14**) 

Yellow solid; mp 78-80 °C; UV λ_max_ 233, 289, 323 nm; IR (KBr) ν_max_ 3058, 2927, 1598, 1467, 1336, 808 cm^−1^; ^1^H NMR (CDCl_3_) δ_H_ 3.96 (2H, t, *J* = 7.9 Hz, H-3), 2.94 (2H, t, *J* = 8.3 Hz, H-4), 7.67 (1H, d, overlap, H-5), 7.20 (1H, m, H-6), 7.28 (1H, m, H-7), 7.38 (1H, d, overlap, H-8), 7.43 (1H, d, overlap,H-1′), 7.85 (1H, d, overlap, H-2′), 8.47 (1H, s, H-4′), 7.41 (1H, d, overlap, H-5′), 7.50 (1H, m, H-6′), 7.22 (1H, m, H-7′), 8.02 (1H, d, 7.7, H-8′), 4.23 (2H, t, *J* = 7.0 Hz, H-1″), 1.83 (2H, m, H-2″), 1.28-1.33 (14H, m, H-4″~H-9″), 0.93 (t, 6.3, H-10″); ^13^C NMR (CDCl_3_) δ_C_ 159.9 (s, C-1), 48.1 (t, C-3), 19.2 (t, C-4), 117.9 (s, C-4a), 125.4 (s, C-4b), 119.8 (d, C-5), 120.1 (d, C-6), 124.3 (d, C-7), 112.1 (d, C-8), 136.7 (s, C-8a), 128.2 (s, C-9a), 108.8 (d, C-1′), 125.7 (d, C-2′), 141.3 (s, C-3′), 120.6 (d, C-4′), 122.7 (s, C-4′a), 122.7 (s, C-4′b), 108.7 (d, C-5′), 125.9 (d, C-6′), 119.1 (d, C-7′), 120.3 (d, C-8′), 127.8 (s, C-8′a), 140.7 (s, C-9′a), 43.0 (t, C-1″), 28.7 (t, C-2″), 27.1 (t, C-3″), 29.1-29.6 (t, C-4″~C-7″), 31.7 (t, C-8″), 22.5 (t, C-9″), 14.0 (q, C-10″); EIMS *m*/*z* 475 (39, M^+^), 460 (4), 349 (27), 335 (25), 335 (63), 319 (10), 173 (76), 159 (22), 97 (19), 83 (23), 69 (38), 57 (79); HREIMS *m*/*z *475.2987 ([M]^+^, calcd. for C_33_H_37_N_3_, 475.2988)_._

3.3.8. 1-(9′-Hexadecyl-3′-carbazolyl)-3,4-dihydro-β-carboline (**16**) 

Yellow solid; mp 90-92 °C; UV λ_max_ 238, 288 nm; IR (KBr) ν_max_ 3060, 2925, 1597, 1468, 1338, 744 cm^−1^; ^1^H NMR (CDCl_3_) δ_H_ 3.93 (2H, t, *J* = 8.1 Hz, H-3), 2.93 (2H, t, *J* = 8.1 Hz, H-4), 7.64 (1H, d, overlap, H-5), 7.26 (1H, m, H-6), 7.32 (1H, m, H-7), 7.52 (1H, d, overlap, H-8), 7.38 (1H, d, overlap, H-1′), 7.86 (1H, d, overlap, H-2′), 8.49 (1H, s, H-4′), 7.41 (1H, d, overlap, H-5′), 7.45 (1H, m, H-6′), 7.23 (1H, m, H-7′), 8.04 (1H, d, *J* = 7.6 Hz, H-8′), 4.19 (2H, t, *J* = 7.2 Hz, H-1″), 1.80 (2H, m, H-2″), 1.12-1.46 (26H, m, H-3″~15″), 0.90 (3H, t, *J* = 6.6 Hz, H-16″); ^13^C NMR (CDCl_3_) δ_C_ 160.1 (s, C-1), 47.5 (t, C-3), 19.4 (t, C-4), 112.9 (s, C-4a), 125.4 (s, C-4b), 120.0 (d, C-5), 120.4 (d, C-6), 125.4 (d, C-7), 112.4 (d, C-8), 137.3 (s, C-8a), 127.8 (s, C-9a), 108.9 (d, C-1′), 126.1 (d, C-2′), 141.7 (s, C-3′), 120.7 (d, C-4′), 122.7 (s, C-4′a), 122.9 (s, C-4′b), 109.0 (d, C-5′), 126.1 (d, C-6′), 119.4 (d, C-7′), 120.4 (d, C-8′), 124.9 (s, C-8′a), 140.7 (s, C-9′a), 43.1 (t, C-1″), 22.6-31.9 (C-2″~15″), 14.1 (q, C-16″); EIMS *m*/*z* 559 (20, M^+^), 532 (3), 418 (2), 346 (20), 180 (6), 174 (30), 83 (5), 69 (11), 57 (41), 44 (100); HRESIMS *m*/*z* 560.4008 ([M + H]^+^, calcd. for C_39_H_50_N_3_, 560.4005).

3.3.9. 1-(9′-Eicosyl-3′-carbazolyl)-3,4-dihydro-β-carboline (**18**) 

Yellow solid; mp 91-93 °C; UV λ_max_ 222, 239, 260, 288 nm; IR (KBr) ν_max_ 3062, 2921, 1465, 1338, 744 cm^−1^; ^1^H NMR (CDCl_3_) δ_H_ 3.87 (2H, t, *J* = 8.0 Hz, H-3), 2.93 (2H, t, *J* = 8.10 Hz, H-4), 7.61 (1H, d, overlap, H-5), 7.16 (1H, m, H-6), 7.33 (1H, m, H-7), 7.53 (1H, d, overlap, H-8), 7.29 (1H, d, overlap,H-1′), 7.99 (1H, d, overlap, H-2′), 8.70 (1H, s, H-4′), 7.38 (1H, d, overlap, H-5′), 7.44 (1H, m, H-6′), 7.18 (1H, m, H-7′), 8.08 (1H, d, *J* = 7.3 Hz, H-8′), 4.07 (2H, t, *J* = 7.3 Hz, H-1″), 1.75 (2H, m, H-2″), 1.25-1.37 (34H, m, H-3″~19″), 0.88 (3H, t, *J* = 6.7 Hz, H-20″); ^13^C NMR (CDCl_3_) δ_C_ 160.6 (s, C-1), 44.8 (t, C-3), 19.2 (t, C-4), 113.2 (s, C-4a), 127.3 (s, C-4b), 120.3 (d, C-5), 120.9 (d, C-6), 126.4 (d, C-7), 113.2 (d, C-8), 139.2 (s, C-8a), 127.3 (s, C-9a), 109.1 (d, C-1′), 127.3 (d, C-2′), 142.4 (s, C-3′), 122.1 (d, C-4′), 122.6 (s, C-4′a), 123.0 (s, C-4′b), 109.1 (d, C-5′), 126.4 (d, C-6′), 119.9 (d, C-7′), 121.1 (d, C-8′), 126.4 (s, C-8′a), 140.8 (s, C-9′a), 43.0 (t, C-1″), 22.6-31.9 (C-2″~19″), 14.1 (q, C-20″); EIMS *m*/*z* 615 (17, M^+^), 588 (4), 364 (20), 246 (14), 174 (15), 121 (35), 91 (41), 71 (45), 69 (64), 57 (100); HRESIMS *m*/*z* 616.4627 ([M + H]^+^, calcd. for C_43_H_48_N_3_, 616.4631).

### 3.4. Synthesis of Compounds **19**-**22**

To a stirred solution of carbazole (2.0 g, 1.2 mmol) and K_2_CO_3_ (1.7 g) in acetone (30 mL), 1-bromopropane, 1-bromopentane, 1-bromooctane and 1-bromodecane were slowly added, respectively (each 5 mL). The reaction mixture was stirred at 50 °C for 24 hours. After filtration and evaporation of the solvent under vacuum, the residue was chromatographed on a silicagel column (60 g) and eluted with *n*-hexane to afford compounds **19**-**22** with a yield which varied in a range of 23-25%.

#### 3.4.1. *N*-Propylcarbazole (**19**)

White solid; UV λ_max_ 244, 261, 293, 330, 344 nm; IR (CH_2_Cl_2_) ν_max_ 3021, 1596, 1484, 1344, 721 cm^−1^; ^1^H NMR (CDCl_3_) δ_H_ 8.16 (1H, d, *J* = 7.7 Hz, H-2), 7.31 (1H, dd, *J* = 7.7, 6.8 Hz, H-3), 7.52 (1H, dd, *J* = 7.7, 6.8 Hz, H-4), 7.45 (1H, d, *J* = 7.9 Hz, H-5), 4.32 (2H, t, *J* = 7.1 Hz, H-1′), 1.97 (2H, m, H-2′), 1.03 (3H, t, *J* = 7.4 Hz, H-3′); ^13^C NMR (CDCl_3_) δ_C_ 140.3 (s, C-1), 120.1 (d, C-2), 118.8 (d, C-3), 126.5 (d, C-4), 108.9 (d, C-5), 122.6 (s, C-6), 44.1 (t, C-1′), 22.3 (t, C-2′), 11.5 (q, C-3′); EIMS *m*/*z* 209 (85, M^+^), 180 (100), 166 (30), 152 (56), 140 (16), 127 (5), 90 (8), 84 (8), 77 (7), 49 (11).

#### 3.4.2. *N*-Pentylcarbazole (**20**)

White solid; UV λ_max_ 254, 261, 293, 330, 344 nm; IR (CH_2_Cl_2_) ν_max_ 3064, 1596, 1484, 1346, 721 cm^−1^; ^1^H NMR (CDCl_3_) δ_H_ 8.14 (1H, d, *J* = 7.7 Hz, H-2), 7.26 (1H, dd, *J* = 7.7, 6.8 Hz, H-3), 7.50 (1H, dd, *J* = 7.7, 6.8 Hz, H-4), 7.43 (1H, d, *J* = 7.9 Hz, H-5), 4.33 (2H, t, *J* = 7.1 Hz, H-1′), 1.94 (2H, m, H-2′), 1.38-1.43 (4H, overlap, H-3′, 4′), 0.91 (3H, t, *J* = 6.8 Hz, H-5′; ^13^C NMR (CDCl_3_) δ_C_ 140.4 (s, C-1), 120.3 (d, C-2), 118.6 (d, C-3), 125.5 (d, C-4), 108.6 (d, C-5), 122.8 (s, C-6), 43.0 (t, C-1′), 28.6 (t, C-2′), 29.4 (t, C-3′), 22.4 (t, C-4′), 13.9 (q, C-5′); EIMS *m*/*z* 237 (27, M^+^), 180 (100), 166 (7), 152 (19), 140 (5), 127 (2), 84 (2), 77 (2), 49 (2).

#### 3.4.3. *N*-Octylcarbazole (**21**)

White solid; UV λ_max_ 264, 263, 330, 344 nm; IR (CH_2_Cl_2_) ν_max_ 3040, 1596, 1484, 1346, 748, 721 cm^−1^; ^1^H NMR (CDCl_3_) δ_H_ 8.23 (1H, d, *J* = 7.7 Hz, H-2), 7.33 (1H, dd, *J* = 7.7, 6.8 Hz, H-3), 7.58 (1H, dd, *J* = 7.7, 6.8 Hz, H-4), 7.51 (1H, d, *J* = 7.9 Hz, H-5), 4.37 (2H, t, *J* = 7.2 Hz, H-1′), 1.97 (2H, m, H-2′), 1.37-1.44 (10H, overlap, H-3′~7′), 1.01 (3H, t, *J* = 6.2 Hz, H-8′; ^13^C NMR (CDCl_3_) δ_C_ 140.6 (s, C-1), 120.5 (d, C-2), 118.8 (d, C-3), 125.7 (d, C-4), 108.8 (d, C-5), 123.0 (s, C-6), 43.2 (t, C-1′), 29.1 (t, C-2′), 27.4 (t, C-3′), 29.5 (t, C-4′), 29.3 (t, C-5′), 31.9 (t, C-6′), 22.8 (t, C-7′), 14.2 (q, C-8′); EIMS *m*/*z* 279 (84, M^+^), 180 (100), 166 (17), 152 (31), 140 (7), 81 (8), 77 (4), 69 (29), 55 (33).

#### 3.4.4. *N*-Decylcarbazole (**22**)

White solid; UV λ_max_ 263, 330, 344 nm; IR (CH_2_Cl_2_) ν_max_ 2925, 2852, 1484, 1342, 748, 721 cm^−1^; ^1^H NMR (CDCl_3_) δ_H_ 7.96 (1H, d, *J* = 7.7 Hz, H-2), 7.09 (1H, dd, *J* = 7.7, 6.9 Hz, H-3), 7.34 (1H, dd, *J* = 7.7, 6.9 Hz, H-4), 7.24 (1H, d, *J* = 8.0 Hz, H-5), 4.12 (2H, t, *J* = 7.2 Hz, H-1′), 1.71 (2H, m, H-2′), 1.11-1.19 (14H, overlap, H-3′~9′), 0.76 (3H, t, *J* = 6.4 Hz, H-10′; ^13^C NMR (CDCl_3_) δ_C_ 140.3 (s, C-1), 120.2 (d, C-2), 118.6 (d, C-3), 125.4 (d, C-4), 108.5 (d, C-5), 122.8 (s, C-6), 42.9 (t, C-1′), 28.8 (t, C-2′), 27.2 (t, C-3′), 29.2-29.5 (t, C-4′~7′), 31.8 (t, C-8′), 22.6 (t, C-9′), 14.0 (q, C-10′); EIMS *m*/*z* 307 (84, M+), 194 (7), 180 (100), 166 (14), 152 (22), 140 (5), 81 (5), 69 (23), 55 (32).

### 3.5. Synthesis of Compounds **23-26**

To a stirred solution of carbazole (1.0 g, 0.6 mmol) and KOH (0.5 g) in EtOH (20 mL), 1-bromo-3-methylbuane, 2-bromopentane, 1-bromohexadecane, 1-bromoeicosane were slowly added, respectively (each 1 mL or 1 g). The reaction mixture was stirred at 50 °C for 24 h. After filtration and evaporation of the solvent under vacuum, the residue was chromatographed on a silica gel column (30 g) and eluted with *n*-hexane to afford compounds **2** **3**-**2** **6** with a yield which varied in a range of 50-75%.

#### 3.5.1. *N*-3′-Methylbutylcarbazole (**23**)

White solid; UV λ_max_ 221, 294, 331, 345 nm; IR (CH_2_Cl_2_) ν_max_ 3053, 2956, 1452, 748, 721 cm^−1^; ^1^H NMR (CDCl_3_) δ_H_ 8.14 (1H, d, *J* = 7.7 Hz, H-2), 7.26 (1H, dd, *J* = 7.7, 7.0 Hz, H-3), 7.49 (1H, dd, *J* = 7.9, 7.0 Hz, H-4), 7.43 (1H, d, *J* = 7.9 Hz, H-5), 4.34 (2H, t, *J* = 7.4 Hz, H-1′), 1.77 (4H, m, H-2′, 3′), 1.06 (4H, d, *J* = 6.2 Hz, H-4′, 5′); ^13^C NMR (CDCl_3_) δ_C_ 140.3 (s, C-1), 120.3 (d, C-2), 118.7 (d, C-3), 125.5 (d, C-4), 108.5 (d, C-5), 122.9 (s, C-6), 41.3 (t, C-1′), 26.1 (t, C-2′), 37.5 (d, C-3′), 22.6 (q, C-4′), 22.6 (q, C-5′); EIMS *m*/*z* 237 (94, M^+^), 180 (100), 166 (20), 152 (39), 140 (15), 127 (4), 69 (5), 41 (69).

#### 3.5.2. *N*-1′-Methylpropylcarbazole (**24**)

White solid; UV λ_max_ 221, 294, 330, 344 nm; IR (CH_2_Cl_2_) ν_max_ 2962, 1454, 748, 721 cm^−1^; ^1^H NMR (CDCl_3_) δ_H_ 8.19 (1H, d, *J* = 7.7 Hz, H-2), 7.29 (1H, dd, *J* = 7.7, 6.9 Hz, H-3), 7.50 (1H, dd, *J* = 8.0, 6.9 Hz, H-4), 7.58 (1H, d, *J* = 8.0 Hz, H-5), 4.74 (2H, m, H-1′), 1.74 (2H, d, *J* = 7.0 Hz, H-2′), 2.07, (2H, m, H-3′), 0.86 (3H, t, *J* = 7.4 Hz, H-4′); ^13^C NMR (CDCl^3^) δ_C_ 139.9 (s, C-1), 120.2 (d, C-2), 118.5 (d, C-3), 125.3 (d, C-4), 110.0 (d, C-5), 123.2 (s, C-6), 52.8 (d, C-1′), 19.0 (q, C-2′), 27.9 (t, C-3′), 11.4 (q, C-4′); EIMS *m*/*z* 223 (36, M^+^), 194 (100), 180 (4), 166 (36), 152 (6), 140 (21), 115 (7), 57 (13), 49 (8).

#### 3.5.3. *N*-Hexadecylcarbazole (**25**)

White solid; UV λ_max_ 229, 236, 261, 293 nm; IR (CH_2_Cl_2_) ν_max_ 3049, 2943, 1452, 746, 719 cm^−1^; ^1^H NMR (CDCl_3_) δ_H_ 8.11 (1H, d, *J* = 7.8 Hz, H-2), 7.24 (1H, dd, *J* = 7.8, 6.9 Hz, H-3), 7.47 (1H, dd, *J* = 8.0, 6.9 Hz, H-4), 7.42 (1H, d, *J* = 8.0 Hz, H-5), 4.31 (2H, t, *J* = 7.2 Hz, H-1′), 1.88 (2H, m, H-2′), 1.25-1.27 (26H, overlap, H-3′~15′), 0.89 (3H, t, *J* = 6.3 Hz, H-16′); ^13^C NMR (CDCl_3_) δ_C_ 140.2 (s, C-1), 120.3 (d, C-2), 118.6 (d, C-3), 125.6 (d, C-4), 108.7 (d, C-5), 122.8 (s, C-6), 43.2 (t, C-1′), 22.8-32.0 (t, C-2′~15′), 14.2 (q, C-16′); EIMS *m*/*z* 391 (82, M^+^), 194 (7), 180 (100), 167 (9), 152 (10), 97 (11), 83 (18), 69 (30), 57 (51), 43 (68).

#### 3.5.4. *N*-Eicosylcarbazole (**26**)

White solid; UV λ_max_ 333, 346 nm; IR (CH_2_Cl_2_) ν_max_ 3050, 2956, 1450, 746, 719 cm^−1^; ^1^H NMR (CDCl_3_) δ_H_ 8.25 (1H, d, *J* = 7.7 Hz, H-2), 7.37 (1H, t, *J* = 7.4 Hz, H-3), 7.60 (1H, t, *J* = 7.4 Hz, H-4), 7.52 (1H, d, *J* = 8.5 Hz, H-5), 4.38 (2H, t, *J* = 7.2 Hz, H-1′), 1.98 (2H, m, H-2′), 1.40-1.44 (34H, overlap, H-3′~19′), 1.07 (3H, t, *J* = 6.4 Hz, H-16′); ^13^C NMR (CDCl_3_) δ_C_ 140.4 (s, C-1), 120.3 (d, C-2), 118.7 (d, C-3), 125.5 (d, C-4), 108.6 (d, C-5), 122.9 (s, C-6), 43.0 (t, C-1′), 22.7-32.0 (t, C-2′~19′), 14.1 (q, C-20′); EIMS *m*/*z* 447 (69, M^+^), 194 (5), 180 (100), 167 (8), 152 (7), 97 (3), 83 (5), 69 (9), 57 (33), 43 (60).

### 3.6. Synthesis of Compounds **27-30**

To a stirred solution of *N*-propylcarbazole (**19**, 620 mg, 2.9 mmol), *N*-pentylcarbazole (**20**, 680 mg, 2.8 mmol), *N*-octylcarbazole (**2** **1**, 1 g, 3.5 mmol), or *N*-decylcarbazole (**2** **2**, 1 g, 3.2 mmol), with hexamethylenetetramine (420-500 mg, 3-3.5 mmol) in tetrahydrofuran (3-10 mL), trifluroacetic acid (1-3.5 mL) was slowly added. The reaction mixtures were refluxed at 80 °C for 3.5 h. After cooling, the solution was partitioned between H_2_O and CHCl_3_. The CHCl_3_-soluble layer was evaporated under vacuum, the residue was chromatographed on a silica gel column (10 g) and eluted with *N*-hexane/CHCl_3_ mixture with increasing polarity to yield compounds **27**-**30** with a yield 30-34%. 

#### 3.6.1. *N*-Propyl-3-carbazolyl Carboxyaldehyde (**27**)

White solid; UV λ_max_ 234, 271, 329 nm; IR (CH_2_Cl_2_) ν_max_ 2964, 2875, 1685, 1592, 1469, 1349, 1338, 806 cm^-1^; ^1^H NMR (CDCl_3_) δ_H_ 7.45 (1H, d, *J* = 8.4 Hz, H-1), 7.98 (1H, d, *J* = 8.5 Hz, H-2), 8.60 (1H, s, H-4), 7.47 (1H, d, *J* = 6.2 Hz, H-5), 7.53 (1H, dd, *J* = 7.1, 6.2 Hz, H-6), 7.33 (1H, dd, *J* = 7.7, 7.1 Hz, H-7), 8.15 (1H, d, *J* = 7.7 Hz, H-8), 10.09 (s, CHO), 4.29 (2H, t, *J* = 7.1 Hz, H-1′), 1.93 (2H, m, H-2′), 0.98 (3H, t, *J* = 7.4 Hz, H-3′); ^13^C NMR (CDCl_3_) δ_C_ 109.0 (d, C-1), 127.2 (d, C-2), 128.5 (s, C-3), 124.0 (d, C-4), 123.1 (s, C-4a), 123.0 (s, C-4b), 109.5 (d, C-5), 126.7 (d, C-6), 120.3 (d, C-7), 120.7 (d, C-8), 141.3 (s, C-8a), 144.2 (s, C-9a), 191.8 (d, CHO), 44.9 (t, C-1′), 22.3 (t, C-2′), 11.8 (q, C-3′); EIMS *m*/*z* 237 (84, M^+^), 208 (100), 180 (24), 166 (12), 152 (21), 139 (7), 84 (7), 77 (3), 69 (5), 49 (15).

#### 3.6.2. *N*-Pentyl-3-carbazolyl Carboxyaldehyde (**28**)

White solid; UV λ_max_ 234, 271, 329 nm; IR (CH_2_Cl_2_) ν_max_ 2954, 2929, 1685, 1592, 1467, 1349, 1326, 748 cm^−1^; ^1^H NMR (CDCl_3_) δ_H_ 7.47 (1H, d, *J* = 8.4 Hz, H-1), 8.01 (1H, d, *J* = 8.5 Hz, H-2), 8.61 (1H, s, H-4), 7.50 (1H, d, *J* = 6.0 Hz, H-5), 7.55 (1H, dd, *J* = 7.0, 6.0 Hz, H-6), 7.33 (1H, dd, *J* = 7.6, 7.0 Hz, H-7), 8.16 (1H, d, *J* = 7.6 Hz, H-8), 10.10 (s, CHO), 4.34 (2H, t, *J* = 7.3 Hz, H-1′), 1.89 (2H, m, H-2′), 1.26-1.37 (2H, m, H-3′), 1.26-1.37 (2H, m, H-4′), 0.98 (3H, t, *J* = 6.3 Hz, H-5′); ^13^C NMR (CDCl_3_) δ_C_ 109.3 (d, C-1), 127.1 (d, C-2), 128.4 (s, C-3), 123.9 (d, C-4), 122.9 (s, C-4a), 123.0 (s, C-4b), 108.9 (d, C-5), 126.6 (d, C-6), 120.2 (d, C-7), 120.7 (d, C-8), 141.1 (s, C-8a), 144.0 (s, C-9a), 191.7 (d, CHO), 43.3 (t, C-1′), 28.5 (t, C-2′), 29.3 (t, C-3′), 22.3 (t, C-4′), 13.8 (q, C-5′); EIMS *m*/*z* 266 (99, M + 1^+^), 265 (76, M^+^), 208 (34), 180 (14), 167 (12), 154 (100), 136 (73), 107 (25), 89 (21), 77 (25), 55 (26).

#### 3.6.3. *N*-Octyl-3-carbazolyl Carboxyaldehyde (**29**)

White solid; UV λ_max_ 234, 276, 290, 331 nm; IR (CH_2_Cl_2_) ν_max_ 2950, 2927, 2875, 1687, 1592, 1467, 1351, 1338 cm^−1^; ^1^H NMR (CDCl_3_) δ_H_ 7.46 (1H, d, *J* = 8.4 Hz, H-1), 8.02 (1H, d, *J* = 8.5 Hz, H-2), 8.62 (1H, s, H-4), 7.48 (1H, d, *J* = 7.7 Hz, H-5), 7.54 (1H, dd, *J* = 7.1, 7.7 Hz, H-6), 7.33 (1H, dd, *J* = 7.6, 7.1 Hz, H-7), 8.16 (1H, d, *J* = 7.6 Hz, H-8), 10.10 (s, CHO), 4.34 (2H, t, *J* = 7.1 Hz, H-1′), 1.88 (2H, m, H-2′), 1.25-1.36 (2H, m, H-3′), 1.25-1.30 (2H, m, H-4′), 1.26-1.36 (2H, m, H-5′), 1.25-1.36 (2H, m, H-6′), 1.25-1.36 (2H, m, H-7′), 0.86 (3H, t, *J* = 7.4 Hz, H-8′); ^13^C NMR (CDCl_3_) δ_C_ 109.0 (d, C-1), 127.2 (d, C-2), 128.5 (s, C-3), 124.0 (d, C-4), 123.1 (s, C-4a), 123.0 (s, C-4b), 109.4 (d, C-5), 126.7 (d, C-6), 120.3 (d, C-7), 120.8 (d, C-8), 141.2 (s, C-8a), 144.1 (s, C-9a), 191.8 (d, CHO), 43.5 (t, C-1′), 29.1 (t, C-2′), 27.3 (t, C-3′), 29.7 (t, C-4′), 29.3 (t, C-5′), 31.8 (t, C-6′), 22.6 (t, C-7′), 14.1 (q, C-8′); EIMS *m*/*z* 307 (71, M^+^), 242 (12), 224 (56), 208 (100), 180 (34), 166 (13), 152 (25), 139 (6), 77 (4), 69 (26), 55 (43).

#### 3.6.4. *N*-Decyl-3-carbazolyl Carboxyaldehyde (**30**)

White solid; UV λ_max_ 234, 274, 290, 331 nm; IR (CH_2_Cl_2_) ν_max_ 2952, 2925, 1687, 1592, 1467, 1351, 1338, 806 cm^−1^; ^1^H NMR (CDCl_3_) δ_H_ 7.47 (1H, d, *J* = 8.4 Hz, H-1), 8.02 (1H, d, *J* = 8.4 Hz, H-2), 8.62 (1H, s, H-4), 7.49 (1H, d, *J* = 7.5 Hz, H-5), 7.54 (1H, dd, *J* = 6.9, 7.5 Hz, H-6), 7.35 (1H, dd, *J* = 7.6, 6.9 Hz, H-7), 8.17 (1H, d, *J* = 7.6 Hz, H-8), 10.11 (s, CHO), 4.31 (2H, t, *J* = 7.2 Hz, H-1′), 1.91 (2H, m, H-2′), 1.26-1.37 (2H, m, H-3′), 1.26-1.37 (2H, m, H-4′), 1.26-1.37 (2H, m, H-5′), 1.26-1.37 (2H, m, H-6′), 1.26-1.37 (2H, m, H-7′), 1.26-1.37 (2H, m, H-8′), 1.26-1.37 (2H, m, H-9′), 0.89 (3H, t, *J* = 6.2 Hz, H-10′); ^13^C NMR (CDCl_3_) δ_C_ 109.0 (d, C-1), 127.2 (d, C-2), 128.5 (s, C-3), 124.0 (d, C-4), 123.1 (s, C-4a), 123.0 (s, C-4b), 109.4 (d, C-5), 126.7 (d, C-6), 120.3 (d, C-7), 120.8 (d, C-8), 141.2 (s, C-8a), 144.1 (s, C-9a), 191.8 (d, CHO), 43.5 (t, C-1′), 28.9 (t, C-2′), 27.3 (t, C-3′), 29.3-29.5 (t, C-4′), 29.3-29.5 (t, C-5′), 29.3-29.5 (t, C-6′), 29.3-29.5 (t, C-7′), 31.9 (t, C-8′). 22.7 (t, C-9′), 14.1 (q, C-10′); EIMS *m*/*z* 335 (67, M^+^), 224 (7), 208 (100), 194 (5), 180 (23), 166 (7), 77 (1), 69 (9), 55 (3), 43 (43).

### 3.7. Synthesis of Compounds **31-34**

Trifluroacetic acid (2.5-5 mL) was slowly added to a stirred solution of *N*-3′-methylbutylcarbazole (**23**, 950 mg, 5.1 mmol), *N*-1′-methylpropylcarbazole (**2** **4**, 950 mg, 4.3 mmol), *N*-hexadecylcarbazole (**2** **5**, 1.2 g, 3.0 mmol), or *N*-decylcarbazole (**2** **6**, 950 mg, 2.1 mmol), with hexamethylenetetramine (810 mg-1 g, 5.1-6.3 mmol) in tetrahydrofuran (2.5-5 mL). The reaction mixtures were refluxed at 80 °C for 3.5 hours. After cooling, the solution was partitioned between H_2_O and CHCl_3_. The CHCl_3_-soluble layer was evaporated under vacuum, the residue was chromatographed on a silica gel column (10 g) and eluted with *N*-hexane/CHCl_3_ mixture with increasing polarity to yield compounds **31**-**3** **4** with a yield of 21-25%. 

#### 3.7.1. *N*-3′-Methylbutyl-3-carbazolyl Carboxyaldehyde (**31**)

White solid; UV λ_max_ 232, 271, 294, 331 nm; IR (CH_2_Cl_2_) ν_max_ 2956, 1685, 1593, 748 cm^−1^; ^1^H NMR (CDCl_3_) δ_H_ 7.45 (1H, d, *J* = 8.3 Hz, H-1), 8.01 (1H, d, *J* = 8.5 Hz, H-2), 8.60 (1H, s, H-4), 7.45 (1H, d, *J* = 8.0 Hz, H-5), 7.54 (1H, dd, *J* = 8.0Hz, H-6), 7.33 (1H, dd, *J* = 7.2, 7.7 Hz, H-7), 8.15 (1H, d, *J* = 7.7 Hz, H-8), 10.10 (s, CHO), 4.33 (2H, t, *J* = 6.6 Hz, H-1′), 1.76 (2H, m, H-2′), 1.76 (3H, m, H-3′), 1.04 (2H, d, *J* = 5.0 Hz, H-4′), 1.04 (2H, d, *J* = 5.0 Hz, H-3′); ^13^C NMR (CDCl_3_) δ_C_ 108.8 (d, C-1), 127.1 (d, C-2), 128.5 (s, C-3), 123.9 (d, C-4), 123.0 (s, C-4a), 123.1 (s, C-4b), 109.2 (d, C-5), 126.7 (d, C-6), 120.3 (d, C-7), 120.7 (d, C-8), 141.0 (s, C-8a), 143.9 (s, C-9a), 191.7 (d, CHO), 41.7 (t, C-1′), 26.1 (t, C-2′), 37.4 (d, C-3′), 22.5 (q, C-4′), 22.5 (q, C-5′); EIMS *m*/*z* 265 (81, M^+^), 208 (100), 195 (9), 180 (34), 166 (16), 152 (33), 139 (9), 84 (8), 69 (10), 41 (33).

#### 3.7.2. *N*-1′-Methylpropyl-3-carbazolyl Carboxyaldehyde (**32**)

White solid; UV λ_max_ 232, 276, 294, 332 nm; IR (CH_2_Cl_2_) ν_max_ 2962, 1685, 1684, 1591, 1489, 1338, 748 cm^−1^; ^1^H NMR (CDCl_3_) δ_H_ 7.58 (1H, d, *J* = 8.7 Hz, H-1), 8.00 (1H, d, *J* = 8.7 Hz, H-2), 8.64 (1H, s, H-4), 7.58 (1H, d, *J* = 8.2 Hz, H-5), 7.51 (1H, dd, *J* = 8.2, 7.1 Hz, H-6), 7.32 (1H, dd, *J* = 7.61, 7.7 Hz, H-7), 8.18 (1H, d, *J* = 7.7 Hz, H-8), 10.10 (s, CHO), 4.73 (2H, m, H-1′), 1.71 (2H, d,*J* = 7.0 Hz, H-2′), 2.50-2.30 (2H, m, H-3′), 0.80 (2H, m, H-4′); ^13^C NMR (CDCl_3_) δ_C_ 110.1 (d, C-1), 126.8 (d, C-2), 128.2 (s, C-3), 123.7 (d, C-4), 123.4 (s, C-4a), 123.4 (s, C-4b), 110.8 (d, C-5), 126.4 (d, C-6), 120.0 (d, C-7), 120.6 (d, C-8), 141.0 (s, C-8a), 144.0 (s, C-9a), 191.6 (d, CHO), 53.5 (d, C-1′), 19.1 (q, C-2′), 28.0 (t, C-3′), 11.4 (q, C-4′); EIMS *m*/*z* 251 (80, M^+^), 222 (100), 194 (78), 180 (5), 166 (54), 152 (10), 139 (31), 113 (6), 69 (11), 57 (29).

#### 3.7.3. *N*-Hexadecyl-3-carbazolyl Carboxyaldehyde (**33**)

White solid; UV λ_max_ 234, 294, 332 nm; IR (CH_2_Cl_2_) ν_max_ 2920, 1689, 1593, 1462, 1352, 806 cm^−1^; ^1^H NMR (CDCl_3_) δ_H_ 7.48 (1H, d, *J* = 8.5 Hz, H-1), 8.01 (1H, d, *J* = 8.5 Hz, H-2), 8.62 (1H, s, H-4), 7.46 (1H, d, *J* = 8.1 Hz, H-5), 7.54 (1H, dd, *J* = 8.1, 7.1 Hz, H-6), 7.33 (1H, dd, *J* = 7.1, 7.7 Hz, H-7), 8.17 (1H, d, *J* = 7.7 Hz, H-8), 10.10 (s, CHO), 4.34 (2H, t, *J* = 7.2 Hz, H-1′), 1.90 (2H, m, H-2′), 1.24-1.26 (2H, m, H-3′), 1.24-1.26 (2H, m, H-4′), 1.24-1.26 (2H, m, H-5′), 1.24-1.26 (2H, m, H-6′~15′), 0.89 (2H, m, H-16′); ^13^C NMR (CDCl_3_) δ_C_ 109.0 (d, C-1), 127.2 (d, C-2), 128.6 (s, C-3), 126.8 (d, C-4), 123.1 (s, C-4a), 123.1 (s, C-4b), 109.5 (d, C-5), 126.8 (d, C-6), 120.3 (d, C-7), 120.8 (d, C-8), 141.2 (s, C-8a), 144.1 (s, C-9a), 191.8 (d, CHO), 43.5 (t, C-1′), 22.8-32.0 (m, C-2′), 22.8-32.0 (m, C-3′), 22.8-32.0 (m, C-4′), 22.8-32.0 (m, C-5′), 22.8-32.0 (m, C-6′~15′), 14.2 (q, C-16′); EIMS *m*/*z* 419 (53, M^+^), 208 (100), 194 (4), 180 (20), 166 (6), 152 (6), 83 (2), 69 (7), 57 (31), 43 (91).

#### 3.7.4. *N*-Eicosyl-3-carbazolyl Carboxyaldehyde (**34**)

White solid; UV λ_max_ 235, 275, 290 nm; IR (CH_2_Cl_2_) ν_max_ 2918, 1687, 752 cm^−1^; ^1^H NMR (CDCl_3_) δ_H_ 7.46 (1H, d, *J* = 8.5 Hz, H-1), 8.01 (1H, d, *J* = 8.5 Hz, H-2), 8.60 (1H, s, H-4), 7.46 (1H, d, *J* = 8.1 Hz, H-5), 7.54 (1H, dd, *J* = 8.1, 7.1 Hz, H-6), 7.33 (1H, dd, *J* = 7.1, 7.7 Hz, H-7), 8.17 (1H, d, *J* = 7.7 Hz, H-8), 10.10 (s, CHO), 4.31 (2H, t, *J* = 7.2 Hz, H-1′), 1.89 (2H, m, H-2′), 1.25-1.27 (2H, m, H-3′), 1.25-1.27 (2H, m, H-4′), 1.25-1.27 (2H, m, H-5′), 1.25-1.27 (2H, m, H-6′~15′), 1.25-1.27 (2H, m, H-16′), 1.25-1.27 (2H, m, H-17′~19′), 0.90 (3H, t, *J* = 7.7 Hz, H-20′); ^13^C NMR (CDCl_3_) δ_C_ 108.9 (d, C-1), 127.1 (d, C-2), 128.8 (s, C-3), 123.9 (d, C-4), 123.0 (s, C-4a), 123.0 (s, C-4b), 109.3 (d, C-5), 126.6 (d, C-6), 120.2 (d, C-7), 120.7 (d, C-8), 140.6 (s, C-8a), 143.6 (s, C-9a), 191.6 (d, CHO), 43.4 (t, C-1′), 22.7-31.9 (t, C-2′), 22.7-31.9 (t, C-3′), 22.7-31.9 (t, C-4′), 22.7-31.9 (t, C-5′), 22.7-31.9 (t, C-6′~15′), 22.7-31.9 (t, C-16′), 22.7-31.9 (t, C-17′~19′), 14.1 (q, C-20′).; EIMS *m*/*z* 475 (71, M^+^), 208 (100), 194 (5), 180 (28), 166 (7), 152 (8), 83 (7), 69 (17), 57 (57), 43 (88)

### 3.8. Cytotoxicity Assay

The cytotoxic activities of compounds against KB (human mouth epidermoid carcinoma), DLD (human colon adenocarcinoma), NCI-H661 (human lung large cell carcinoma), Hepa (human hepatoma), and HepG2/A2 (human hepatoblastoma) cells were assayed by the MTT{3-(4,5-dimethylthiazole-2-yl)-2,5-diphenyltetrazolium bromide} colorimetric assay as previously described [23]. The cells for assay were cultured in RPMI-1640 medium supplemented with 5% CO_2_ in an incubator at 37 °C. The cytotoxicity assay depends on the binding of methylene blue to fixed monolayers of cells at pH 8.5, washing the monolayer, and releasing the dye by lowering the pH value. Samples and control standard drugs were prepared at a concentration of 1, 10, 40, and 100 μg/mL. After seeding 2880 cells/well in a 96-well microplate for 3 h, 20 μL of sample or standard agent was placed in each well and incubated at 37 °C for 3 days. After removing the medium from the microplates, the cells were fixed with 10% formaldehyde in 0.9% saline for 30 min, then dyed with 1% (w/v) methylene blue in 0.01 M borate-buffer (100 μL/well) for 30 min. The 96-well plate was dipped into a 0.01 M borate-buffer solution four times in order to remove the dye. Then, 100 μL/well of EtOH-0.1 M HCl (1:1) was added as a dye eluting solvent, and the absorbance was measured on a microtiter plate reader (Dynatech, MR 7000) at a wavelength of 650 nm. The ED_50_ value was defined by a comparison with the untreated cells as the concentration of test sample resulting in 50% reduction of absorbance. Doxorubicin was used as a standard compound.

### 3.9. Relaxation Assay of Topoisomerases I and II

Topoisomerases I and II **(**topo I and II) assays were measured by assessing relaxation of supercoiled pBR322 plasmid DNA according to [24]. Using camptothecin (CPT) as topo I and etoposide (VP-16) as topo II positive controls, test samples were dissolved in 5% (v/v) DMSO and then diluted to appropriate concentrations. In summary, topo I (TopoGen) was mixed with the test sample and 10° volume of assay buffer (100 mM Tris-HCl, 10 mM EDTA, 1.5 M NACl, 1.0% BSA, 1 M spermidine, and 50% glycerol), and then supercoiled DNA (pBR322) was added. In topo II assay, the mixture contained test sample and buffer including 50 mM Tris-HCl, 120 mM KCl, 10 mM MgCl_2_, 0.5 mM ATP, 0.5 mM dithiothreitol, 2 μg BSA, pBR322 plasmid DNA (0.25 μg), and 3U of topo II (TopoGen) in final volume of 20 μL. After incubation of topo I or topo II mixture for 30 min at 37 °C, 2 λ 10% SDS and 2.5 λ proteinase K were added for 1 h. The reaction mixtures were electrophoresed on a 2% agarose gel (50 V, 20 min; 100 V, 30 min; 110 V, 30 min) and stained with ethidium bromide. Finally, by a densitometer of ImageMaster^®^ (Fujifilm thermal imaging system, FTI-500), the gels were directly scanned and the area representing supercoiled DNA was calculated. Concentrations for 50% inhibition (IC_50_) were determined by interpolation from plots of topoisomerases I or II activity *versus* inhibitor concentration. Etoposide was used as a standard.

### 3.10. Molecular Modeling

The three dimensional structures of DNA duplexes were obtained from the Brookhaven Protein Databank (PDB code 108d or 264d). The 3D structures of compound **2** were constructed as a protonated form and were assigned Gasteiger-Huckel partial charges using Sybyl 7.0 (Tripos Associates; St. Louis, MO, USA) as previously described [25]. The AutoTors module was used to specify two rotatable bonds in **2** for AutoDock. The initial complex structure was then optimized by energy minimization with the Tripos force field, employing the Powell method with an energy-gradient-convergence criterion of 0.05 kcal/(mol Ǻ) and a distance-dependent dielectric constant of 4 r. To carry out AutoDock simulations, a grid box was defined to enclose the interaction cavity with dimensions of 18.8 × 18.8 × 18.8 Ǻ. The grid maps for energy scoring were calculated with Autogrid using a grid-point spacing of 0.375 Ǻ, and 100 × 76 × 76 grid points for 108 d, or 70 × 70 × 110 grid points for 264 d. 200 independent docking runs were carried out using the Lamarckian genetic algorithm (LGA) with a maximum number of 5,000,000 energy evaluations and a population of 50 randomly initiated individuals. The lowest-energy docked conformations proposed the energetically favorable binding modes of compound **2** to DNA.

## 4. Conclusions

In summary, a series of 1-substituted carbazolyl-1,2,3,4-tetrahydro- and carbazolyl-3,4-dihydro-β-carboline analogs have been synthesized and evaluated as potential antitumor agents. Among them, compound **7** and **6** showed the most potent and selective activity against NCI-H661 and KB tumor cells, respectively. Compound **9** possessed most potent activity against DLD tumor cells. Compound **2** exhibited most promising activity against KB, NCI-H661 and Hepa (or HepG_2_/A_2_) tumor cell lines. Inhibition of human DNA topoisomerase II revealed that compounds **3** and **4** are quite promising for further development of enzyme inhibitors. The SAR revealed that there was a lack of complete correlation between carbon numbers of the side chain and biological activities. However, the negative correlation was present between the alkyl side chain length in cytotoxicity tests. The optimal chain length is between 2 and 5 carbons. The optimal chain length for anti-topoisomerase II may be 3 and 5 carbons. The DNA binding capacity, which favored minor groove binding, was thus influenced by the alkyl substitution on carbazole chromophore and proton donor on β-carboline chromophore. On the basis of the SAR study, synthesis of analogs of lead compounds **2**, **3**, **6**, **7** and **9**, in order to search for more potent activity, is currently in progress.

## References

[1] Blackman A.J., Matthews D.J., Narkowicz C.K. (1987). β-Carboline alkaloids from the marine bryozoan *Costaticella hastata*. J. Nat. Prod..

[2] Kearns P.S., Coll J.C., Rideout J.A. (1995). A beta-carboline dimer from an ascidian, *Didemnum* sp. J. Nat. Prod..

[3] Kobayashi J., Tsuda M., Kawasaki N., Sasaki T., Mikami Y. (1994). 6-Hydroxymanzamine A and 3,4-Dihydromanzamine A, New Alkaloids from the Okinawan Marine Sponge *Amphimedon* sp. J. Nat. Prod..

[4] Mc Nulty J., Still I.W.J. (1995). Selective methylation and Stereoselective Reduction of a β-Carboline to a N(2)-Methyl-1,2,3,4-tetrahydro-β-carboline. Tetrahedron Lett..

[5] Still I.W.J., Mc Nulty J. (1989). The Synthesis of Eudistomins S and T: β-carbolines from the Tunicate *Eudistoma olivaceum*. Heterocycles.

[6] Nowak W., Gerlach H. (1993). Synthesis of Manzamine C, Infractine and 6-Hydroxyinfractine. Liebigs Ann. Chem..

[7] Badre A., Boulanger A., Mansom A.E., Banaigs B., Combaut G., Francisco C. (1994). Eudistomin U and Isoeudistomin U, New Alkaloids from the Carribean Ascidian *Lissoclinum fragile*. J. Nat. Prod..

[8] Rinehart K.L., Kobajashi J., Harbour G.C., Gilmore J., Mascal M., Holt T.G., Shield L.S., Lafargue F. (1987). Eudistomins A–Q, β-carbolines from the antiviral Caribbean tunicate Eudistoma olivaceum. J. Am. Chem. Soc..

[9] Crews P., Cheng X.C., Adamczeski M., Rodriguez J., Jaspars M., Schmitz F.J., Traeger S.C., Pordesimo E.O. (1994). 1,2,3,4-tetrahydro-8-hydroxymanzamines, alkaloids from two different haplosclerid sponges. Tetrahedron.

[10] Sakai R., Kohmoto S., Higa T., Jefford C.W., Bernardinelli G. (1987). Manzamine B and C, two novel alkaloids from the sponge *haliclona* sp. Tetrahedron Lett..

[11] Munro M.H.G., Luibrand R.T., Blunt J.W., Scheuer P.J. (1987). The search for antiviral and anticancer compounds from marine organisms. Bioorganic Marine Chemistry.

[12] Ichiba T., Sakai R., Kohmoto S., Saucy G., Higa T. (1988). New manzamine alkaloids from a sponge of the genus xestospongia. Tetrahedron Lett..

[13] Higa T., Atta-Ur-Rahman Ed. (1989). Structure elucidation. Studies in Natural Product Chemistry.

[14] Yousaf M., Hammond N.L., Peng J., Wahyuono S., McIntosh K.A., Charman W.N., Mayer A.M.S., Hamann M.T. (2004). New Manzamine Alkaloids from an Indo-Pacific Sponge. Pharmacokinetics, Oral Availability, and the Significant Activity of Several Manzamines against HIV-I, AIDS Opportunistic Infections, and Inflammatory Diseases. J. Med. Chem..

[15] Sakai R., Higa T., Jefford C.W., Bernardinelli G. (1986). Manzamine A, a novel antitumor alkaloid from a sponge. J. Am. Chem. Soc..

[16] Shen Y.C., Tai H.R., Duh C.Y. (1996). Bioactive constituents from *Heliclona* sp, a Formosan marine sponge. Chin. Pharm. J..

[17] Yeh S.F., Shen Y.C. (2004). 1-Substituted 1,2,3,4-Tetrahydro-β-Carboline and 3,4-Dihydro-β-Carboline and Analogs as Antitumor. Agents.US Patent.

[18] Duff J.C.  (1945). A new method for the preparation ofp-dialkylaminobenzaldehydes. J. Chem. Soc..

[19] Valentine D., Scot J.W. (1978). Asymmetric synthesis. Synthesis.

[20] Kawashima Y., Horiguchi A., Taguchi M., Tuyuki Y., Karasawa Y., Araki H., Hatayama K. (1995). Synthesis and Pharmacological Evaluation of 1,2,3,4-Tetrahydro-β-carboline Derivatives. Chem. Pharm. Bull..

[21] Kondo K., Shigemori H., Kikuchi Y., Ishibashi M., Sasaki T., Kobajashi J. (1992). Ircinals A and B from the Okinawan marine spongeIrciniasp.: plausible biogenetic precursors of manzamine alkaloids. J. Org. Chem.

[22] Pezzuto J.M., Che C.T., Mc Pherson D.D., Zhu J.P., Topcu G., Erdelmeir C.A.J., Cordell G.A. (1991). DNA as an Affinity Probe Useful in the Detection and Isolation of Biologically Active Natural Products. J. Nat. Prod..

[23] Shen Y.-C., Wang S.-S., Pan Y.-L., Lo K.-L., Chakraborty R., Chien C.T., Kuo Y.H., Lin Y.-C. (2002). New Taxane Diterpenoids from the Leaves and Twigs of *Taxus sumatrana*. J. Nat. Prod..

[24] Yang Kuo L.M., Chen K.Y., Hwang S.Y., Chen J. L., Liu Y.Y., Liaw C.C., Ye P.H., Chou C.J., Shen C.C., Kuo Y.H. (2005). DNA Topoisomerase I Inhibitor , Ergosterol Peroxide from *Penicillium oxalicum*. Planta Med..

[25] Tu L.-C., Chen C.-S., Hsiao I.-C., Chern J.-W., Lin C.-H., Shen Y.-C., Yeh S.-F. (2005). The β-Carboline Analog Mana-Hox Causes Mitotic Aberration by Interacting with DNA. Chem. Biol..

